# Microbial Biotransformation Products and Pathways of Dichloroacetamide Herbicide Safeners

**DOI:** 10.1021/acs.estlett.2c00862

**Published:** 2022-12-05

**Authors:** Monica E. McFadden, Keith P. Reber, John D. Sivey, David M. Cwiertny, Gregory H. LeFevre

**Affiliations:** † Department of Civil and Environmental Engineering, University of Iowa, 4105 Seamans Center for the Engineering Arts and Sciences, Iowa City, Iowa 52242, United States; ‡ IIHR-Hydroscience and Engineering, University of Iowa, 100 C. Maxwell Stanley Hydraulics Laboratory, Iowa City, Iowa 52242, United States; § Department of Chemistry, Towson University, Towson, Maryland 21252, United States; ⊥ Center for Health Effects of Environmental Contamination (CHEEC), University of Iowa, 251 North Capitol St., Chemistry Building, Room W195, Iowa City, Iowa 52242, United States; ∥ Public Policy Center, University of Iowa, 310 South Grand Ave., 209 South Quadrangle, Iowa City, Iowa 52242, United States

**Keywords:** Safeners, microbial biotransformation, cometabolism, biodegradation, dechlorination, benoxacor, dichlormid, CDAA

## Abstract

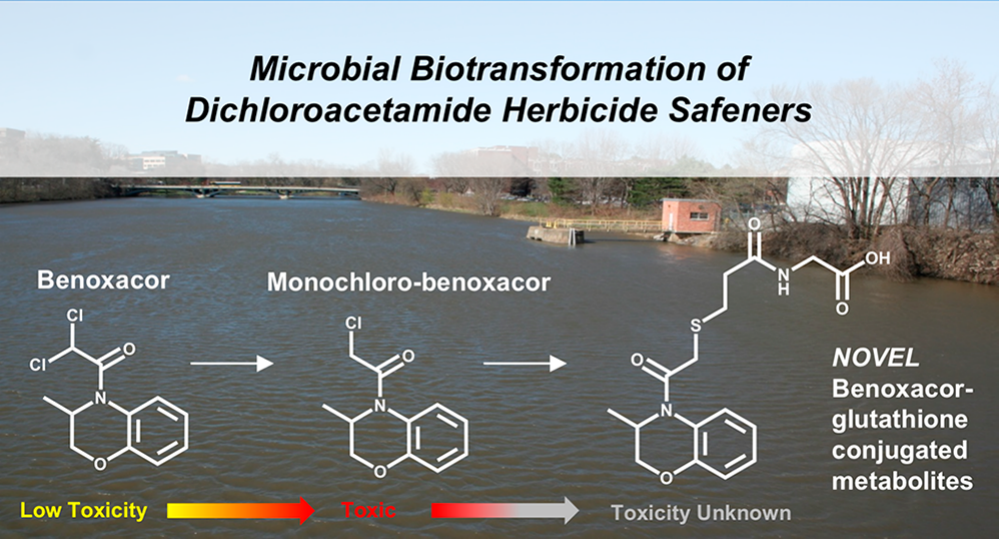

Dichloroacetamide safeners are common ingredients in commercial herbicide formulations. We previously investigated the environmental fate of dichloroacetamides via photolysis and hydrolysis, but other potentially important, environmentally relevant fate processes remain uncharacterized and may yield products of concern. Here, we examined microbial biotransformation of two dichloroacetamide safeners, benoxacor and dichlormid, to identify products and elucidate pathways. Using aerobic microcosms inoculated with river sediment, we demonstrated that microbial biotransformations of benoxacor and dichlormid proceed primarily, if not exclusively, via cometabolism. Benoxacor was transformed by both hydrolysis and microbial biotransformation processes; in most cases, biotransformation rates were faster than hydrolysis rates. We identified multiple novel products of benoxacor and dichlormid not previously observed for microbial processes, with several products similar to those reported for structurally related chloroacetamide herbicides, thus indicating potential for conserved biotransformation mechanisms across both chemical classes. Observed products include monochlorinated species such as the banned herbicide CDAA (from dichlormid), glutathione conjugates, and sulfur-containing species. We propose a transformation pathway wherein benoxacor and dichlormid are first dechlorinated, likely via microbial hydrolysis, and subsequently conjugated with glutathione. This is the first study reporting biological dechlorination of dichloroacetamides to yield monochlorinated products in aerobic environments.

## Introduction

Dichloroacetamide safeners are coapplied with chloroacetamide herbicides to selectively protect crops from unintended herbicide toxicity.^1−4^ Due to their extensive use (>8 × 10^6^ kg/year globally) and hydrophilic nature, the four most common dichloroacetamides (AD-67, benoxacor, dichlormid, and furilazole) have been detected in surface waters throughout the midwestern U.S., yet their environmental fates remain largely underinvestigated.^3,5−11^ Existing research, including studies by our groups, indicates that safeners can transform in the environment to yield products with increased biological activity and, in some cases, increased toxicity.^7,11,12^ For example, dichloroacetamides in iron-rich anaerobic environments can undergo reductive dechlorination to yield more toxic products, including formation of CDAA (known as allidochlor or 2-chloro-N,N-bis(prop-2-enyl)acetamide; an herbicide banned in the United States due in part to human health concerns) from dichlormid, as well as monochloro-benoxacor (toxic toward insect larvae; LOEC = 0.1 mg kg^–1^) from benoxacor.^7,11,12^ Recently, we probed dichloroacetamides' environmental fate, focusing on photolysis and hydrolysis.^13,14^ Only benoxacor transformed by direct photolysis, and hydrolysis rates were slow and only environmentally relevant under basic (pH 10–11) conditions.^13,14^ Thus, there are potentially significant environmental fate processes relevant to dichloroacetamide safeners, notably microbial biotransformation, that remain uncharacterized and may yield transformation products of concern.

Microbial biotransformation is likely relevant because dichloroacetamide safeners are biologically active, and their structurally related chloroacetamide herbicide coformulants undergo microbial biotransformation.^3,15−18^ Dichloroacetamide safeners are designed to activate crop defense genes to promote conjugation of herbicides to glutathione (GSH), yielding conjugates of lessened phytotoxicity.^1,2,4,19^ However, some GSH conjugates also have been identified for dichlormid, benoxacor, and furilazole safeners in plants.^19−21^ Further, microbial biotransformation reactions are considered among the most important processes controlling the fates of chloroacetamide herbicides in the environment; metabolites can account for up to 99% of a chloroacetamide herbicide’s measured concentration in surface and groundwater.^15,22−27^ The major products of chloroacetamides are the highly mobile, generally nontoxic ethanesulfonic acid (ESA) and oxanilic acid (OA) derivatives of acetochlor, alachlor, metolachlor, and propachlor.^15,16,22,28^ Considering the close structural similarities of chloroacetamide herbicides and dichloroacetamide safeners (the former of which are known to microbially transform), as well as the ability of safeners to be biotransformed by plants via conjugation reactions, we hypothesized there is potential for analogous microbial biotransformation reactions of dichloroacetamide safeners. Thus, dichloroacetamides’ high uses, presence in midwestern surface waters, and potential to yield deleterious products indicate an urgent need to better characterize dichloroacetamide biotransformation.

We report, for the first time, microbial transformation of safeners benoxacor and dichlormid via cometabolic processes. Benoxacor and dichlormid were chosen as representative, environmentally relevant safeners because they have been widely detected in rural surface waters throughout the midwestern U.S. (detection frequencies were 29% and 15% for benoxacor and dichlormid, respectively, with maximum concentrations of 190 ng/L [0.73 nM] and 42 ng/L [0.20 nM], respectively), and they are among the most well studied of the dichloroacetamide safeners. Microbial transformation products include monochlorinated species (e.g., the regulated herbicide CDAA via dichlormid), glutathione conjugates, and sulfur-containing products. Together, these products indicate a general transformation pathway analogous to those reported for microbial transformation of chloroacetamide herbicides.

## Materials and Methods

### Chemicals

Herbicide safeners benoxacor (Sigma-Aldrich) and dichlormid (TCI America) and the herbicide allidochlor (known as CDAA; ChemService) were purchased at >97% purity. Monochloro-benoxacor was synthesized as described in the Supporting Information. Chemicals are fully described in the SI (Sections S1 and S2).

### Experimental Design

#### Biotransformation Batch Experiments

Biotransformation batch experiments were conducted for benoxacor, monochloro-benoxacor, dichlormid, and CDAA, with sodium acetate added to the nutrient media as a primary carbon source (Section S6). Microcosms were assembled in triplicate by aliquoting 10 mL of nutrient media (Section S3) containing of the safener into autoclaved 14.5 mL amber glass vials, allowing sufficient headspace to maintain an aerobic environment (eqs S1–S8, Table S1). Microcosms were inoculated with 0.5 g of river sediment (Section S4). Hydrolysis control vials (pH 7.4) were prepared in triplicate without sediment. Triplicate no safener controls (i.e., controls inoculated with sediments; Section S4, Figure S7) were prepared without safeners to identify any chromatographic peaks from microbial byproducts unrelated to the analytes of interest. We used a matched-pairs experimental design; all experimental and control vials were sampled concurrently, with at least six sampling time points over approximately 4 weeks. Samples (0.5 mL) were collected with a glass syringe, centrifuged at 10,000×*g* for 5 min, and the supernatant was analyzed. Starting concentrations of analytes were between 10 and 100 μM to facilitate quantification by HPLC-DAD. Safener and herbicide concentrations, and any detected products, were monitored at 220 nm with an Agilent 1260 HPLC-DAD system using our previously published methods (Section S7, Tables S3–S5).^13^

#### Product Elucidation

Product structures were determined using a semiuntargeted metabolomics approach wherein experimental systems were compared to hydrolysis controls and no safener controls to identify biotransformation products of safeners (Figure S7). Samples for product elucidation were taken from the same batch experiments described above near maximum product formation, as indicated by HPLC-DAD peak areas. A Q-Exactive hybrid quadrupole Orbitrap mass spectrometer (Thermo Fisher Scientific, Bremen, Germany) was used for accurate mass identification and MS/MS fragmentation. Analyses were conducted in both positive and negative electrospray ionization modes (Section S7), and data were processed and analyzed using XCalibur (Thermo Fisher Scientific, version 4.2.47) and Compound Discoverer (Thermo Fisher Scientific, version 3.1.0.305) softwares. When available, commercial or synthesized reference standards were spiked into samples to aid in metabolite identification. We used the Schymanski framework^29^ to communicate confidence in identifying products.

## Results and Discussion

### Biotransformation as a Significant Transformation Process

We quantified degradation of benoxacor under laboratory conditions to (1) establish that biotransformation was a relevant process compared to abiotic losses and (2) determine if the safeners served as primary carbon sources or degraded via cometabolism. In the presence of a labile primary carbon source, benoxacor transformed via microbial processes over time scales relevant to environmental fate compared to abiotic losses (Figure S8A). Over 31 days under aerobic laboratory batch conditions, 95 ± 3% and 96.9 ± 0.2% of benoxacor (n = 3; ± SD) transformed in microcosms containing sodium acetate and acetonitrile, respectively; thus, the two labile supplemental carbon sources performed similarly. In contrast, over the same time period, only 40.6 ± 0.8% of benoxacor transformed in aqueous systems where benoxacor was the sole carbon source and 48 ± 3% in systems containing humic acid, a more recalcitrant carbon source unlikely to facilitate cometabolism.^30^ Only 39.0 ± 1.3% of the total benoxacor concentration was transformed in abiotic controls, likely due to hydrolysis; the decay followed first-order kinetics at a rate consistent with our prior studies.^14^ Batch tests for the other safeners studied were conducted as described in the presence of acetate. Structurally related chloroacetamides, including acetochlor, alachlor, and propachlor, are also subject to microbial biotransformations primarily by cometabolism.^15,17,18^ These results are presented to demonstrate that biotransformation can occur at rates greater than abiotic losses; however, laboratory batch systems are recognized not to fully represent environmental conditions.

### Identification of Benoxacor Microbial Biotransformation Products

We observed multiple microbial biotransformation products for benoxacor and dichlormid that are analogous to those reported for structurally related chloroacetamide herbicides, indicating the potential for a common biotransformation mechanism across both chemical classes. For benoxacor, three major products were detected (Table 1). Monochloro-benoxacor was identified based on its accurate mass ([M + H]^+^ 226.06263) and chlorine isotope signature (*m*/*z* 226/228) in a 3:1 ratio. A standard addition of a monochloro-benoxacor synthesized reference standard^11^ (Figures S9–S13) confirmed the product identity with Level 1 confidence.^29^ Monochloro-benoxacor has been identified as a product of iron-mediated reductive dechlorination of benoxacor in anaerobic environments^7,31^ and exerts increased toxicity toward insect larvae compared to the parent benoxacor.^11^

**Table 1:**
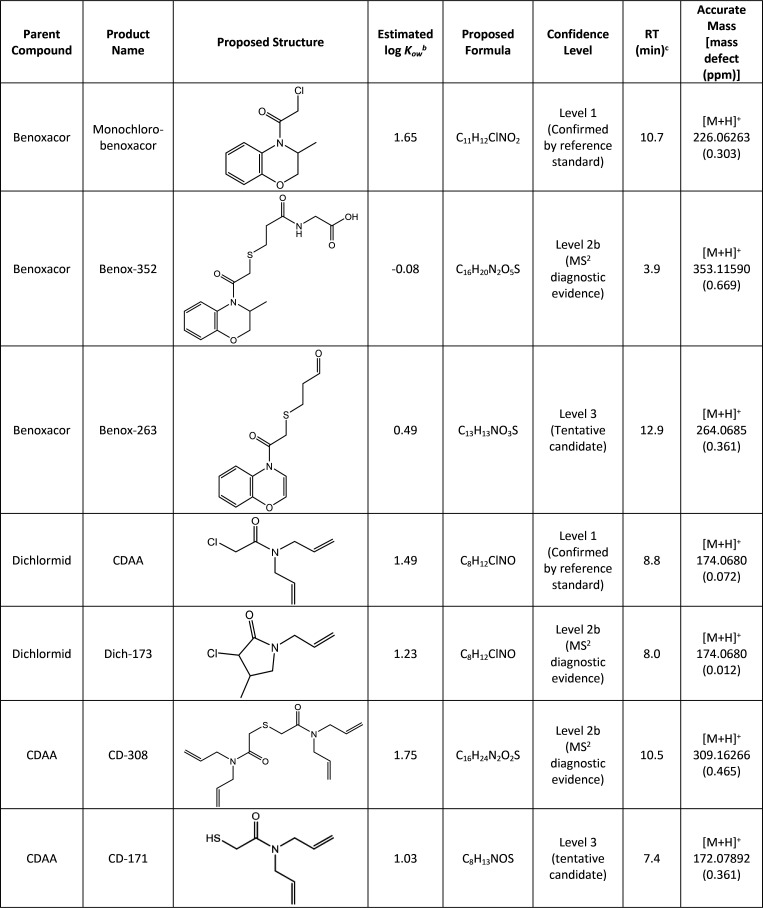
Microbial Biotransformation Products of Benoxacor, Dichlormid, and CDAA^a^

a Confidence level of each product is described according to the framework outlined by Schymanski et al. for identifying small molecules via high resolution mass spectrometry.^29^

b Log *K*_*ow*_ values were estimated by ChemDraw software or obtained from the CompTox dashboard.^39^

c Retention times (RT) correspond to reversed-phase LC-MS/MS experiments. MS data are presented in Figures S10–S23.

**Figure 1:**
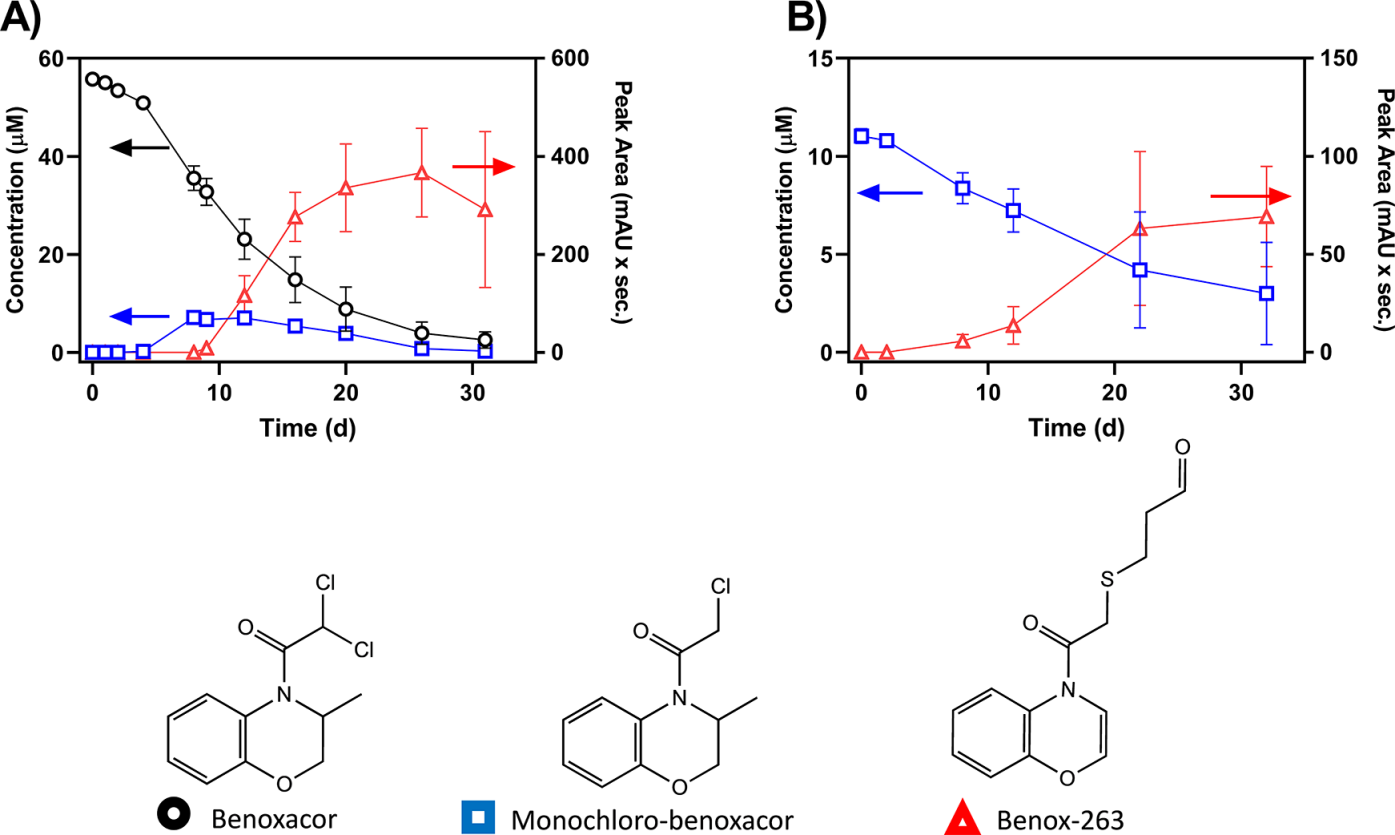
(A) Measured concentrations (benoxacor and monochloro-benoxacor) and peak area (Benox-263) for the microbial biotransformation of benoxacor to yield two major microbial biotransformation products over 31 days. (B) Measured concentration (monochloro-benoxacor) and peak area (Benox-263) for the transformation of monochloro-benoxacor to yield the cysteine-related conjugate Benox-263. Error bars represent one standard deviation (*n* = 3; some error bars are obscured by the data points if very small). Retention times for each compound on the Obritrap mass spectrometer are as follows: benoxacor, 12.1 min; monochloro-benoxacor, 10.7 min; Benox-263, 12.9 min. Descriptions of the transformation products and their confidences in identification for the proposed structures are included in Table 1.

As the benoxacor biotransformation experiment progressed, a second product was detected with an accurate mass [M + H]^+^ of 264.0685 and no observable chlorine isotope signature. Based on the time-dependent trends in monochloro-benoxacor yield (Figure 1A), we suspected that monochloro-benoxacor may degrade to yield this second product, hereafter Benox-263. To probe this pathway for secondary product formation, we conducted the same biotransformation experiment using microcosms containing monochloro-benoxacor (rather than benoxacor) as the starting material. Indeed, HPLC-DAD peak areas for Benox-263 increased concomitantly as the concentration of monochloro-benoxacor decreased (Figure 1B). MS/MS fragmentation patterns indicate replacement of the remaining chlorine of monochloro-benoxacor by a cysteine-related conjugate (Figure S14, Table S12). Further evidence for this structure is provided by minor product peaks of lower molecular weight for which MS/MS data indicate cleavage along the cysteine chain. Several analogous structures have been reported for chloroacetamide herbicide microbial biotransformation products including acetochlor, alachlor, metolachlor, and propachlor; these products and mechanisms are further discussed below.^15,16,22,28^

A third major product of benoxacor biotransformation was not detected by UV–vis spectrophotometry but was identified by Orbitrap LC-MS/MS (Figure S15). The product ([M + H]^+^ 353.11590) occurred both in biotransformation systems where benoxacor was the parent compound (Figure S15) and in systems with monochloro-benoxacor as the starting material. Accurate mass fragmentation, and the presence of numerous minor products with similar fragmentation patterns (Table S12), indicate that Benox-352 is a cysteinyl-glycine (CysGly) conjugate of benoxacor.

These observed products for microbial transformation of benoxacor are consistent with previously published studies on plant metabolism of benoxacor. Miller et al.^20^ proposed pathways for the phytotransformation of benoxacor to a glutathione conjugate in *Zea mays* (maize) cells. The first step involves elimination of one chlorine atom from benoxacor by a glutathione (GSH)-dependent, glutathione S transferase (GST)-catalyzed reaction to yield a resonance-stabilized chlorinated carbanion intermediate (e.g., monochloro-benoxacor) and an electrophilic S-(chloro)GSH conjugate.^20^ One of their observed plant metabolites was a mono-GSH conjugate [4-(glutathione-S-acetyl)-3,4-dihydro-3-methyl-2H-1,4-benzoxazine], which had reported MS/MS fragments that were the same as our microbial metabolites Benox-352 and Benox-263. Although Miller et al. did not directly observe monochloro-benoxacor^20^ and rather implicated it as a pathway intermediate, we detected the formation and decay of monochloro-benoxacor with Level 1 confidence.

Sequential dechlorination and glutathione conjugation under aerobic conditions is an established microbial transformation pathway^16^ for many pesticides, including chloroacetamide herbicides.^15^ These pathways involve intermediates structurally related to those described herein for benoxacor. We thus propose an analogous pathway for benoxacor microbial biotransformation (Scheme S1), which begins with sequential removal of both chlorine atoms and GSH conjugation through a series of GST-mediated reactions.^15,16^ Subsequent removal of γ-glutamic acid would yield a Cys-Gly conjugate (i.e., identical or similar to Benox-352).^15,16^ Carboxypeptidase enzymes are known to cleave the glycine moiety to yield cysteine conjugates, and prior studies demonstrated C-demethylation of a derivative of the chloroacetamide acetochlor in anaerobic sludge reactors.^32,33^ These mechanisms are consistent with products observed herein (e.g., Benox-263).^15,16^ We note that for chloroacetamide herbicides, the reaction proceeds through additional cleavage steps mediated by cysteine β-lyases and subsequent oxidation to yield nontoxic ethanesulfonic acid (ESA) and oxanilic acid (OA) metabolites, which are more mobile and more widely detected than chloroacetamide parent compounds (Table S11).^5,15,16,22,28,34,35^ In our study, however, ESA and OA derivatives were not detected. Mechanistically, microbial dechlorination under aerobic conditions is indeed not a reductive dehalogenation process (e.g., TCE biodegradation under anoxic conditions), but rather a microbial hydrolysis resulting in dechlorination. Compounds with good leaving groups on saturated carbons (e.g., alkyl halides) can transform via enzymatically mediated hydrolysis,^36^ with the thiol group of glutathione serving as a bionucleophile. These enzymatic reactions commonly involve an initial nucleophilic attack (in this case the −S^–^ thiol moiety of GSH) displacing the leaving group (in this case Cl^–^) in an S_N_2 reaction, yielding a GSH adduct. Microbially mediated hydrolysis reactions generally proceed faster than comparable abiotic hydrolysis reactions.^36^

### Identification of Dichlormid Microbial Biotransformation Products

**Figure 2:**
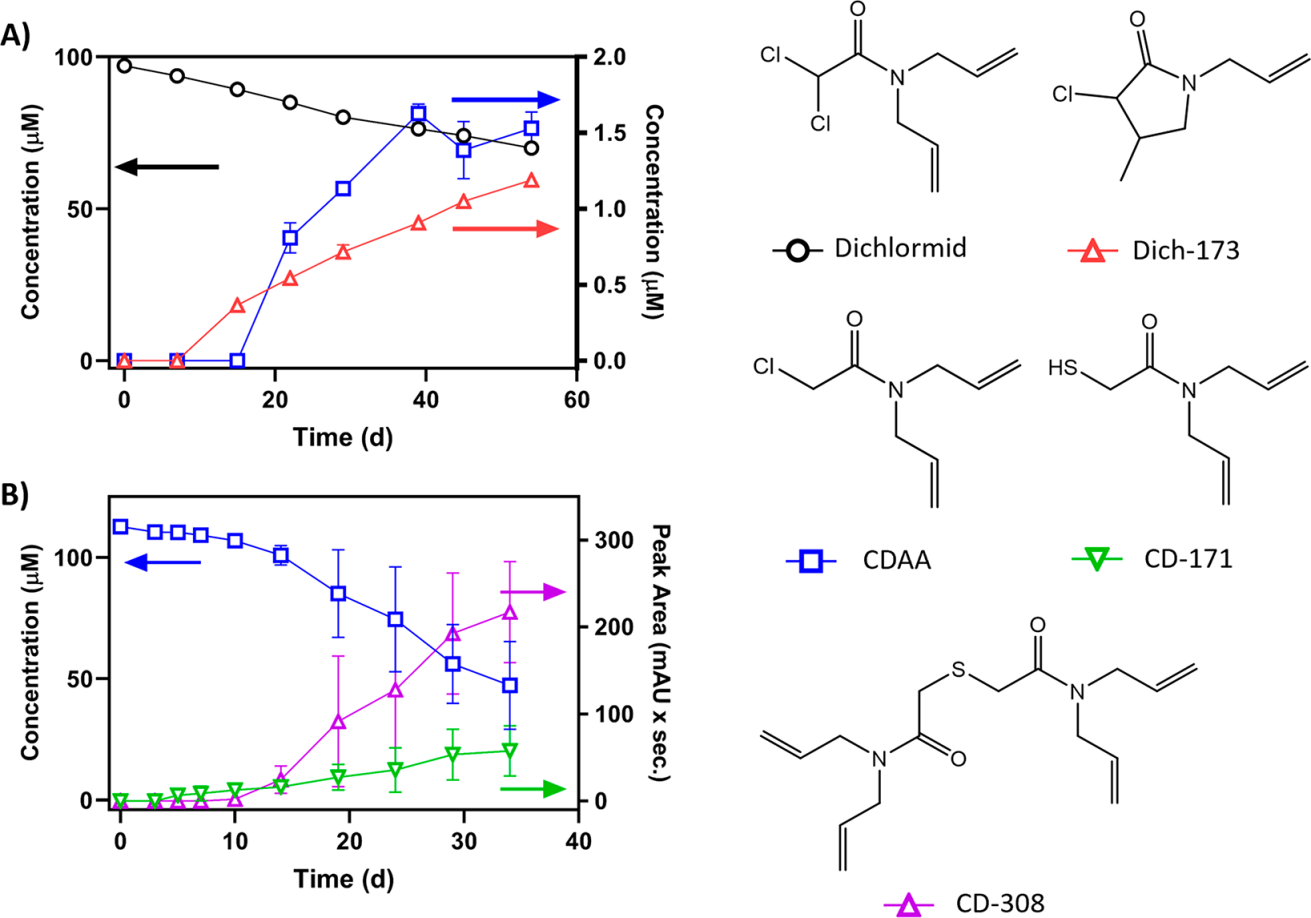
(A) Measured concentration and peak areas for the microbial biotransformation of dichlormid to yield two major monochlorinated microbial biotransformation products over the course of 31 days. The products were identified as the active herbicide CDAA and an isomer (based on data presented by Sivey and Roberts;^7^ Dich-173 is assumed to have the same molar absorptivity as CDAA). (B) Measured concentration and peak area for the transformation of CDAA to yield the proposed sulfur-containing metabolite CD-171 and a dimer (CD-308). Error bars representing one standard deviation (*n* = 3) are present but may be obscured by the data points. Retention times for each compound on the Obritrap mass spectrometer are as follows: dichlormid, 10.6 min; Dich-173, 8.0 min; CDAA, 8.8 min; CD-171, 7.4 min; CD-308, 10.5 min. Descriptions of the transformation products and their confidences in identification for the proposed structures are included in Table 1.

We detected four major microbial biotransformation products from the safener dichlormid (Figures S18–S23). Primary metabolism of dichlormid (Figure 2A) yielded two monochlorinated derivatives. Although the product peaks were distinct (Orbitrap retention times were 8.05 and 8.8 min), they shared an accurate mass [M + H]^+^ 174.0680, consistent with the loss of one chlorine atom. This was supported by the presence of a chlorine isotope signature (*m*/*z* 226/228) in a 3:1 ratio. In iron-rich anaerobic systems, dichlormid can undergo abiotic hydrogenolysis to yield the active herbicide CDAA.^7^ Indeed, standard addition of commercially available CDAA confirmed the structure of the dichlormid product with RT 8.8 min as CDAA to Level 1 confidence. Notably, the pesticide registration for CDAA was canceled by the U.S. EPA in 1984 due to human health concerns.^37^ Thus, microbial biotransformation of dichlormid represents the first instance reported in environmental literature in which a biotransformation process converts an inert safener ingredient into a banned pesticide ingredient with known human health effects. Based on MS/MS fragmentation patterns, the structure of the second monochlorinated metabolite of dichlormid (RT 8.05) was tentatively identified as Dich-173, resulting from CDAA intramolecular cyclization. Sivey and Roberts identified both CDAA (confirmed) and Dich-173 (tentative) as products of abiotic reductive dechlorination.^7^

When CDAA was used as the starting material, we observed two dechlorinated, sulfur-containing products: CD-171 ([M + H]^+^ 172.07892) and dimer CD-308 ([M + H]^+^ 309.16266) (Figure 2B, Table 1). The identification of transformation products that contain sulfur, which was not present in the parent compound, implicates transformation pathways involving the conjugation and cleavage of glutathione.^16^ Control systems lend greater confidence that the incorporation of sulfur into CDAA products was microbially mediated; CD-171 and CD-308 were observed only in the experimental microcosms that contained CDAA and the sediment/microbial inoculum. Prior studies demonstrate that dimerization and trimerization of dichloroacetamide safeners including dichlormid occur readily in the presence of hydrogen sulfide and graphite.^10,38^ Sulfur-substituted products reported herein have similar or greater polarity compared to dichlormid, based on their reversed-phase LC retention times, and are therefore anticipated to be more mobile in aqueous systems relative to the parent safener. Notably, reported concentrations of dichlormid in natural systems are lower than the concentrations of our experimental systems; as such, dimerization of dichlormid products may proceed to a lesser extent.^6^ Nonetheless, a heterogeneous system such as our experimental microcosms may concentrate dichlormid and its products to subsequently promote surface-mediated dimerization,^10^ and this process merits further investigation.

Collectively, we propose that the microbial biotransformation of dichlormid proceeds via the same mechanism as benoxacor, by which a monochlorinated intermediate is conjugated to glutathione and is subsequently cleaved by oxidase enzymes to yield sulfur-containing metabolites.

### Environmental Implications

Microbial biotransformation may be critical to the fate and transformation of dichloroacetamide safeners in the environment because abiotic processes are less relevant. Indeed, our previous work demonstrated only benoxacor transformed by direct photolysis, and hydrolysis rates were slow and only environmentally relevant under basic (pH 10–11) conditions.^13,14^ We described in the Introduction that up to 99% of chloroacetamide herbicide mass has been reported as metabolites. By identifying novel microbial biotransformation products of dichloroacetamide safeners in this work, mass balances of herbicide safeners and their metabolites can be better characterized in environmental samples. Biotransformation products observed in this study have important and urgent implications for water quality and human health; e.g., the herbicide CDAA was banned due to health concerns,^37^ and monochloro-benoxacor is toxic toward insect larvae.^11^ Formation of monochlorinated products of dichloroacetamides under aerobic conditions observed herein is consistent with the literature on aerobic biological dechlorination of pesticides via glutathione-mediated reactions^16^ and contrasts previous reports of CDAA and monochloro-benoxacor formation from dichloroacetamide safeners only in anaerobic iron-rich environments.^7^ Although glutathione conjugation is typically a detoxification mechanism for the organism facilitating the reaction, the biological activity/toxicity of the metabolites reported here have not yet been determined; more ecotoxicity research on safeners and their metabolites released to the surrounding environment is needed, and data from this study can help guide future effects research.

## Supporting Information Available

Chemical and synthesis data, oxygen demand calculations, experimental methods/design, analytical methods, numerical methods, kinetics/rate calculations, numerical results, high-resolution MS spectra, and proposed microbial biotransformation pathway (PDF)
