# Supplementary material for: Microbial Biotransformation Products and Pathways of Dichloroacetamide Herbicide Safeners
Source: Environ Sci Technol Lett. 2022 Dec 5;10(1):72–8. doi: 10.1021/acs.estlett.2c00862 (PMC10111411; doi:10.1021/acs.estlett.2c00862)
Supplement: Supplementary file 1 — ez2c00862_si_001.pdf [file ez2c00862_si_001.pdf]

Supporting Information

Microbial Biotransformation Products and Pathways of Dichloroacetamide Herbicide Safeners

Monica E. McFadden[†,‡], Keith P. Reber[§], John D. Sivey[§], David M. Cwiertny[†,‡,⊥,#], Gregory H. LeFevre*[†,‡],

[†]Department of Civil and Environmental Engineering, University of Iowa, 4105 Seamans Center for the Engineering Arts and Sciences, Iowa City, IA 52242, United States; [‡]IIHR-Hydroscience and Engineering, University of Iowa, 100 C. Maxwell Stanley Hydraulics Laboratory, Iowa City, Iowa, 52242, United States; [§] Department of Chemistry, Towson University, Towson, MD 21252, United States; [⊥] Center for Health Effects of Environmental Contamination (CHEEC), University of Iowa, 251 North Capitol St., Chemistry Building – Room W195, Iowa City, IA 52242, United States; [#] Public Policy Center, University of Iowa, 310 South Grand Ave., 209 South Quadrangle, Iowa City, IA 52242, United States

*Corresponding Author's Contact Information:

Gregory H. LeFevre, Telephone: (319) 335-5655; E-mail: gregory-lefevre@uiowa.edu

CONTAINS: Supplementary Methods and Results; 23 Figures, 12 Tables, 1 Scheme; 37 pages total (inclusive of this page)

Supplementary Methods

Section S1: Chemicals. Chemicals used in bench studies included the dichloroacetamide safeners benoxacor (99.4%, Sigma Aldrich, CAS 98730-04-2, IUPAC 2,2-dichloro-1-(3-methyl-2,3-dihydro-4H-1,4-benzoxazin-4-yl)ethenone) and dichlormid (>97.0%, TCI America, CAS 37764-25-3, IUPAC 2,2-dichloro-N,N-di(prop-2-en-1-yl)acetamide), and the chloroacetamide herbicide allidochlor (known as CDAA; 97%, ChemService, CAS 93-71-0, IUPAC N,N-diallyl-2-chloroacetamide). The microbial biotransformation product monochloro-benoxacor was synthesized and characterized as described below. High-performance liquid chromatography (HPLC) analyses used HPLC-grade acetonitrile (Fisher Scientific) and deionized water purified to 18.2 MΩ•cm. Optima grade acetonitrile and water (Fisher Scientific) were used for liquid chromatography mass spectrometry (LC-MS) analyses. Other chemicals (all ACS certified grade) include: KH_2PO_4 (RPI), K_2HPO_4 (RPI), NH_4Cl (RPI), $Na_2HPO_4•7H_2O$ (Fisher), $MgSO_4•7H_2O$ (Sigma Aldrich), $CaCl_2$ (JT Baker), and $FeCl_2•6H_2O$ (Fisher), which were used in the minimal nutrient media, described below in Section S3.

Section S2: Synthesis of monochloro-benoxacor.

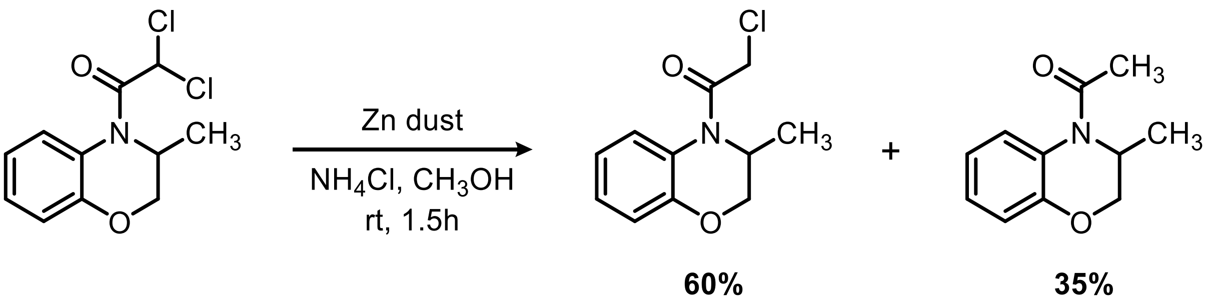

To a 50 mL flask under argon equipped with a magnetic stirring bar was added a solution of benoxacor (150 mg, 0.578 mmol, 1.00 eq.) in 18 mL of methanol. Ammonium chloride (190 mg, 3.55 mmol, 6.15 eq.) was added followed by zinc dust (396 mg, 6.06 mmol, 10.50 eq.), and the heterogeneous reaction mixture was stirred vigorously at room temperature. After 15 minutes, thin

layer chromatography (silica gel stationary phase, 1:1 hexanes / ethyl acetate, UV visualization) showed complete consumption of benoxacor (R_f = 0.74), formation of the *N*-chloroacetyl product (R_f = 0.62), and a small amount of the *N*-acetyl product (R_f = 0.32). After 1.5 hours, thin layer chromatography showed a 2:1 ratio of the two products. The reaction mixture was filtered through Celite to remove the zinc dust, which was then washed with ethyl acetate. The filtrate was concentrated under reduced pressure, and the residue was partitioned between water and ethyl acetate to remove any remaining salts. The organic phase was dried over anhydrous sodium sulfate, the drying agent was removed by filtration, and the solvent was removed under reduced pressure. The crude product mixture was purified by column chromatography on silica gel (2:1 hexanes / ethyl acetate) to afford 78 mg of the *N*-chloroacetyl product (60%) and 39 mg of the *N*-acetyl product (35%) as brown oils.

<u>*N*-chloroacetyl product:</u>

^{1}H NMR (400 MHz, $CDCl_3$): δ 1.21 (3H, br s), 4.17-4.26 (3H, m), 4.35 (1H, d, *J* = 12.3 Hz), 5.02 (1H, br s), 6.87-6.95 (2H, m), 7.06-7.49 (2H, m).

^{13}C NMR (100 MHz, $CDCl_3$): δ 15.3, 41.8, 44.2, 70.1, 117.2, 120.8, 123.0, 124.2, 127.0, 146.4, 164.9.

IR (thin film): ṽ 2976, 2881, 1656, 1585, 1448, 1387, 1325, 1260, 1240, 1207, 1115, 1053, 991, 750, 639 cm^{-1}.

<u>*N*-acetyl product:</u>

^{1}H NMR (400 MHz, $CDCl_3$): δ 1.15 (3H, d, *J* = 6.1 Hz), 2.30 (3H, s), 4.12-4.19 (2H, m), 5.08 (1H, br s), 6.87-6.92 (2H, m), 7.04-7.32 (2H, m).

^{13}C NMR (100 MHz, $CDCl_3$): δ 15.3, 23.3, 43.1, 70.3, 117.0, 120.4, 124.1, 124.9, 126.1, 146.2, 168.8.

IR (thin film): ṽ 2974, 2932, 1655, 1584, 1493, 1374, 1312, 1260, 1123, 986, 796, 750, 611 cm^{-1}.

Note that all NMR signals are broadened due to restricted rotation about the amide bond.

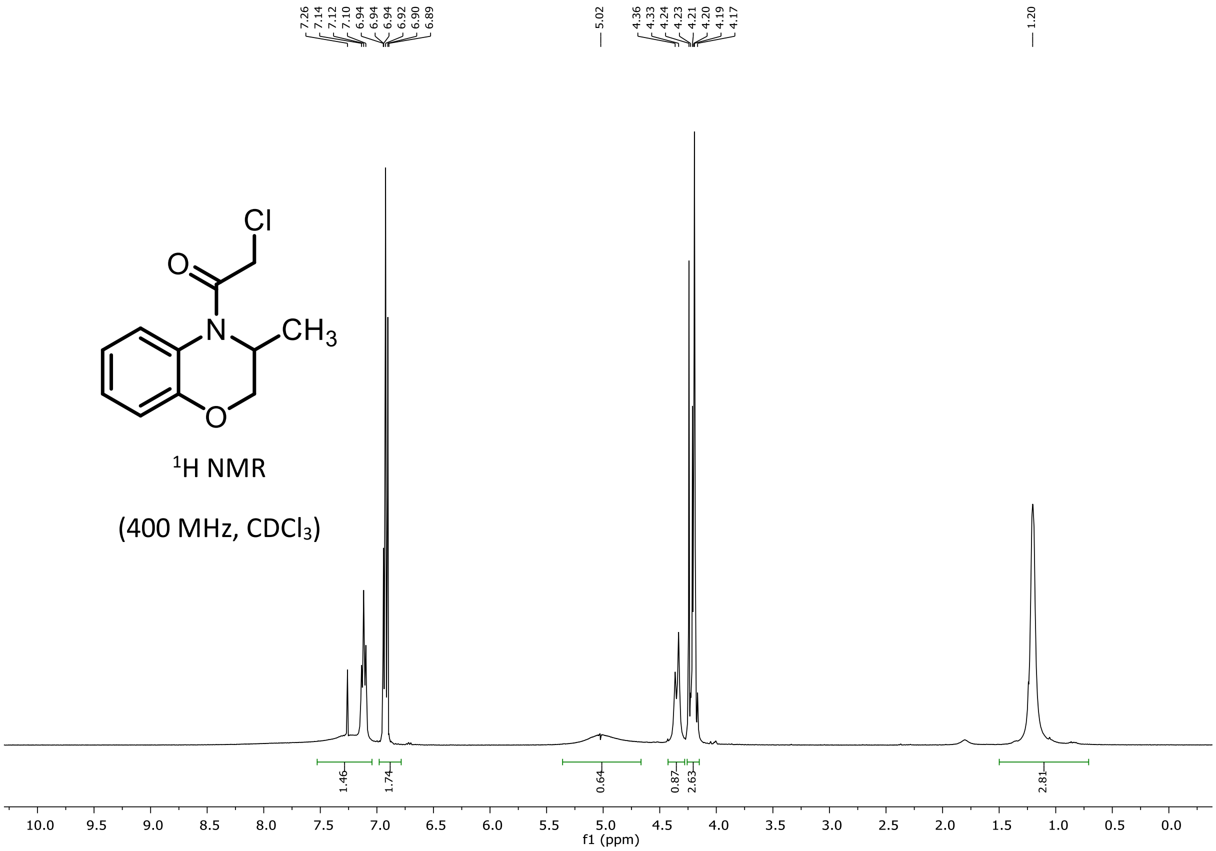

Figure S1: ^{1}H NMR spectrum for the *N*-chloroacetyl product.

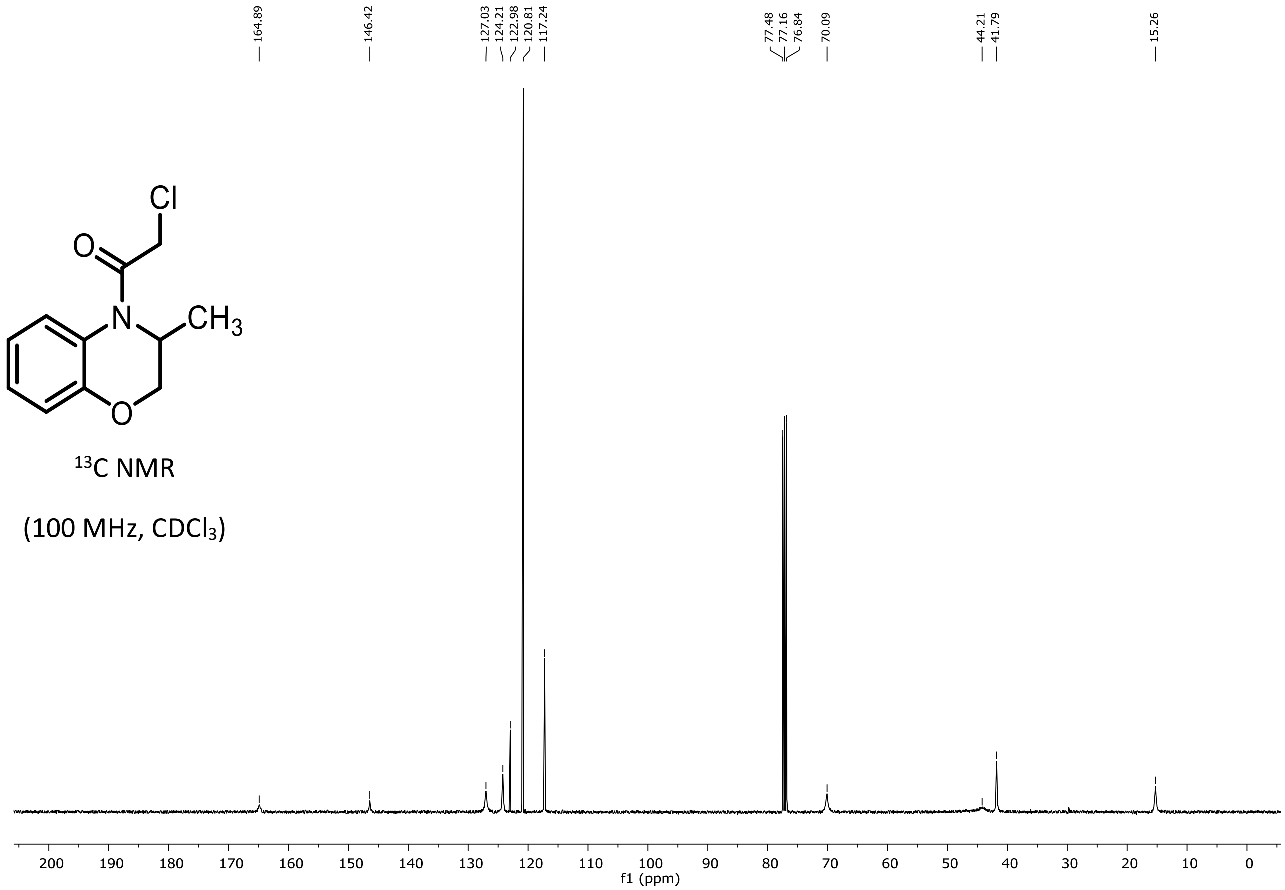

Figure S2: ^{13}C NMR spectrum for the *N*-chloroacetyl product.

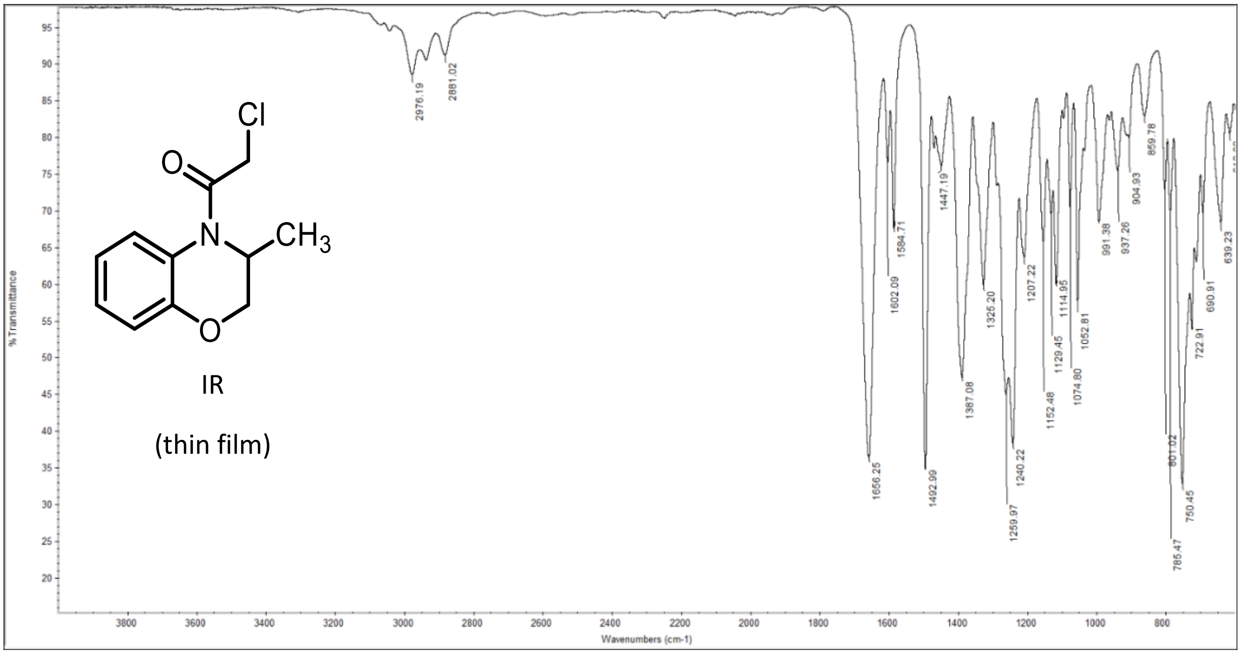

Figure S3: IR (thin film) spectrum for the *N*-chloroacetyl product.

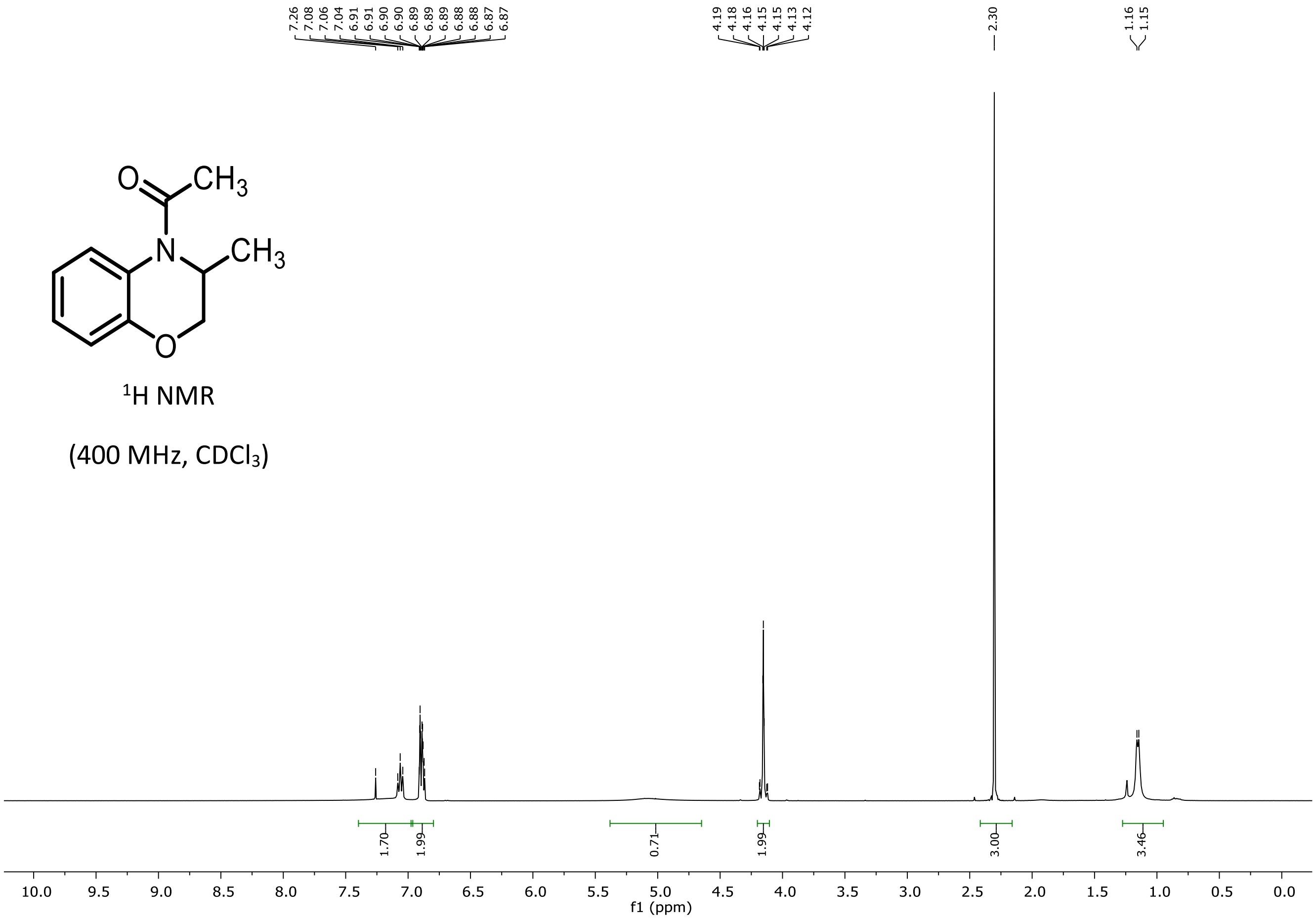

Figure S4: ^{1}H NMR spectrum for the *N*-acetyl product.

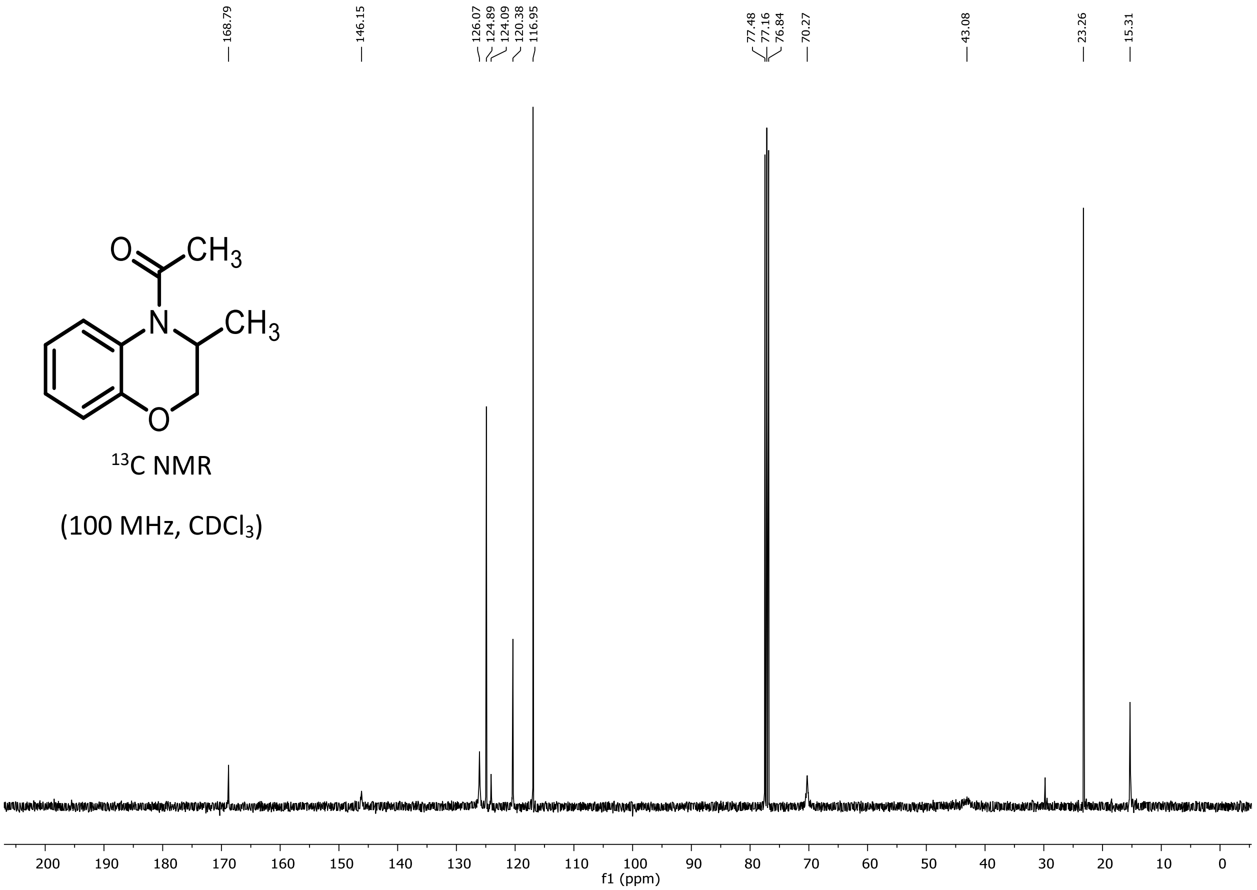

Figure S5: ^{13}C NMR spectrum for the *N*-acetyl product.

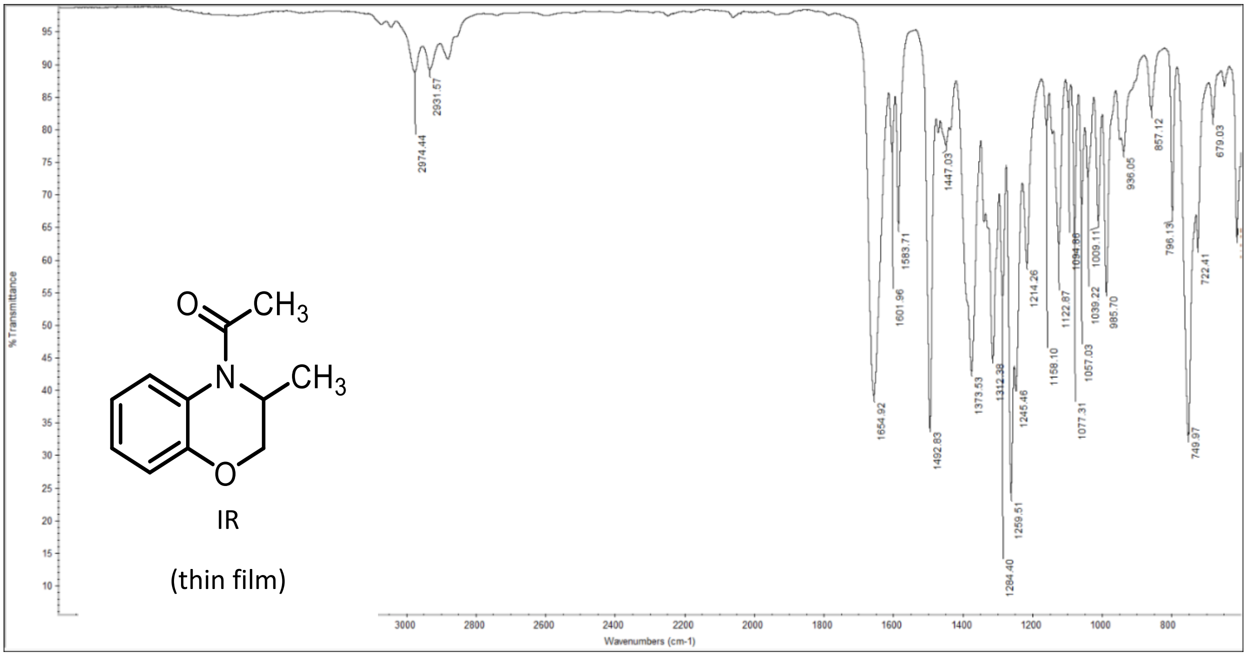

Figure S6: IR (thin film) spectrum for the *N*-acetyl product.

Section S3: Buffered minimal nutrient medium. The nutrient medium used in these studies was originally published by Ghoshal et al.[1]: 170 mg of KH_2PO_4, 435 mg of K_2HPO_4, 850 mg of NH_4Cl, 668 mg of Na_2HPO_4•$7H_2O$, 27.5 mg of $MgSO_4$•$7H_2O$, 27.5 mg of $CaCl_2$, and 0.25 mg of $FeCl_2$•$6H_2O$ were dissolved in 1L of deionized water purified to 18.2 MΩ•cm. The nutrient medium was adjusted to a biologically-relevant pH (7.4 ± 0.1) and filter-sterilized using a 0.2 μm polystyrene bottle-top filter (Corning, Corning, NY). Benoxacor and CDAA were added by dissolving into the nutrient medium. For dichlormid and monochloro-benoxacor, stock solutions that contained 10 mM (dichlormid) or 1 mM (monochloro-benoxacor) prepared in ACN were spiked into the nutrient media to achieve 10 μM concentrations (corresponding to 0.1% and 1.0% of the total volume, respectively.

Section S4: Aerobic microcosm experimental design, setup, and sampling. Microcosms (experimental systems and no-safener controls; Figure S7) were inoculated with 0.5 g of sediment collected from the banks of the Iowa River (Iowa City, IA) during low flow. Sediment

was stored at 2° C and air dried at room temperature prior to weighing (to enable accurate measurement) and was passed through a 2.0 mm sieve.

Slurry samples (0.5 mL) were collected with a glass syringe, centrifuged at 10,000× g for 5 minutes, and the supernatant was analyzed via HPLC.

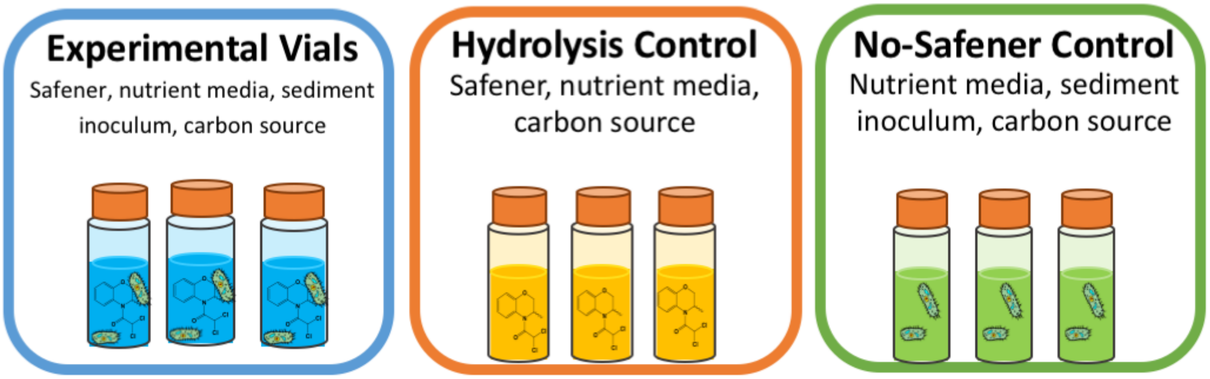

Figure S7: Experimental design of batch microcosms highlighting the experimental systems, hydrolysis controls, and no-safener controls.

Section S5: Headspace calculation

1. *How much O_2 is available in the vial headspace?* We made conservative assumptions about the starting conditions of the microcosms: 1) There is no dissolved oxygen; 2) There is 4.5 mL headspace at the beginning of the experiment; 3) Headspace is 21% O_2; 4) Pressure is 1 atm; 5) System is well-mixed and has adequate O_2 transfer (i.e., no mass transfer limitations that prevent O_2 air and water equilibrium partitioning); 6) We accounted for sediment volume to be subtracted from the available headspace assuming a conservative specific gravity of 1.5 based on Wiesebron et al[2] and assume this conservative based on other work by our laboratory on nearby sediment characterization (Zhi et al[3]). We used the ideal gas law to calculate the mols of oxygen available in the vial headspace:

Estimated volume of sediment: 0.5g sediment / 1.5 g cm^{-3} = 0.33 mL = 3.33×10^{-4} L

$$PV = nRT \quad \textbf{S1}$$

$$(1\ atm)(4.5\ \times\ 10^{-3} - 3.33 \times\ 10^{-4}\ L)(21\%\ O_2) = n\ \left(0.082057 \frac{L \times atm}{K \times mol}\right)(293\ K) \quad \textbf{S2}$$

$$n = 3.64\ \times 10^{-5}\ mol\ O_2 available \quad \textbf{S3}$$

2. *How much O_2 is needed to completely convert all benoxacor to CO_2?* The formula for benoxacor is $C_{11}H_{11}Cl_2NO_2$, so conversion of the carbon in 1 mol of benoxacor to CO_2 would require 11 mol O_2. For a 77 μM (saturated) solution of benoxacor in 10 mL nutrient media:

$$\left(77\ \times 10^{-6} \frac{mol\ benoxacor}{L}\right)(10\ \times 10^{-3}\ L) = 7.7\ \times 10^{-7}\ mol\ benoxacor \quad \textbf{S4}$$

$$(7.7 \times 10^{-7} mol\ benoxacor) \frac{11\ mol\ O_2}{1\ mol\ benoxacor} = 8.47\ \times 10^{-6}\ mol\ O_2 \quad \textbf{S5}$$

3. *How much O_2 is needed to convert all sodium acetate to CO_2?* The formula for sodium acetate is CH_3COONa, so conversion of the carbon in 1 mol of sodium acetate to CO_2 would require 2 mol O_2. The concentration of sodium acetate is 1.57 mg/L, so in 10 mL liquid media in vials we have 1.57 × 10^{-5} g sodium acetate:

$$(1.57\ \times 10^{-5}\ g)\left(\frac{1\ mol}{82.0\ g}\right) = 1.91\ \times 10^{-7}\ mol\ sodium\ acetate \quad \textbf{S6}$$

$$(1.91\ \times 10^{-7}\ mol\ sodium\ acetate)\left(\frac{2\ mol\ O_2}{1\ mol\ sodium\ acetate}\right) = 3.8\ \times 10^{-7}\ mol\ O_2 \quad \textbf{S7}$$

Combining the oxygen demand from sodium acetate and from benoxacor:

$$(8.47\ \times 10^{-6}\ mol\ O_2) +\ 3.8\ \times 10^{-7}\ mol\ O_2 = 8.85\ \times 10^{-6}\ mol\ O_2 \quad \textbf{S8}$$

Thus, there is enough oxygen available in the vials (eqn S3) to maintain an aerobic environment.

Table S1: Oxygen demand for microbial biotransformation systems

Chemicals	Oxygen needed to convert all C to CO_2 (μmol)	O_2 available to maintain aerobic environment? (< 36 μmol)
Benoxacor alone	8.47	yes
Benoxacor with sodium acetate	8.85	yes
Benoxacor with acetonitrile	9.24	yes
Benoxacor with humic acid	8.76	yes
Monochloro-benoxacor in acetonitrile with sodium acetate	1.63	yes
Dichlormid in acetonitrile with sodium acetate	8.39	yes
CDAA with sodium acetate	9.58	yes

Section S6: Co-Metabolism carbon sources

Co-metabolism was initially assessed for benoxacor using minimal nutrient media with either sodium acetate, humic acid, or acetonitrile added as a primary carbon source (Table S2). For co-metabolism studies, labile carbon was added to the minimal nutrient media to achieve 38.3 mM carbon as follows:

Table S2: Co-metabolism carbon sources

Carbon source	Concentration or Volume Added
Sodium Acetate	1.57 mg L^{-1}
Humic Acid	0.87 mg L^{-1}
Acetonitrile	10 mL

For kinetic experiments involving CDAA, a stock solution was prepared in nutrient media without an organic cosolvent. Dichlormid and monochloro-benoxacor were on hand only as

stock solutions in HPLC-grade acetonitrile; for those systems, the nutrient media was spiked with stock solution (corresponding to 0.1–1.0% of the total volume).

Section S7: Analytical methods

Dichloroacetamide samples were quantified using an Agilent 1260 HPLC with diode array detection (HPLC-DAD) and OpenLab ChemStation software (Waldbronn, Germany). HPLC methods generally followed those developed by Kral *et al.*[4] using an Agilent Zorbax Eclipse XDB-C18 column (4.6 × 150 mm, 3.5 μm), acetonitrile and water gradated eluent (**Table S3**) at a flow rate of 1 mL/min, and 25 μL injection volumes. Column temperature was not controlled.[4]

For structure identification of transformation products, samples were analyzed using an Orbitrap Q-Exactive mass spectrometer (Thermo Fisher Scientific, Bremen, Germany) equipped with a heated ESI source. The operating parameters were as follows: sheath gas, auxiliary gas, sweep gas, 58, 16, 3 (arbitrary units) respectively; spray voltage, 3.5 kV; temperature of ion transfer capillary, 281 °C; S-lens RF level, 80. Stepped collision energies for fragmentation were 20, 30, and 40 V. Data were acquired using Full MS/dd-MS^2 (Top N) mode. With this mode, all ions present in the sample were collected in the quadrupole during the first scan event (full MS scan). The second scan event (dd-MS^2) was performed to obtain all fragmented ions from the top five most abundant parent ions identified during the first scan event.

Compound Discoverer analysis used a processing workflow template (‘Environmental with Stats Unknown ID with Online and Local Database Searches’) built for untargeted analysis of environmental samples. The workflow performed retention time alignment, unknown compound detection, and compound grouping across all samples.

Table S3: HPLC mobile phase gradient

Time (min)	% Acetonitrile	% Deionized Water
0	25	75
11	80	20
12	25	75
15	25	75

Table S4: Orbitrap Q-Exactive mobile phase gradient

Time (min)	% Acetonitrile	% Deionized Water
0	25	75
2	25	75
13	90	10
14	25	75
22	25	75

Lower Limit of Detection Calculation:

Table S5: Instrument limit of detection (iLOD) for benoxacor, dichlormid, and their degradates

Compound	iLOD (μM)
Benoxacor	0.031
Monochloro-benoxacor	0.019
Dichlormid	0.074
CDAA	0.041

Section S8: Numerical Methods.

To compare the relative decay among safeners and herbicides, percent biotransformation vs time data were fit using a modified form of the three-parameter[28] Gompertz function:

$$f(t) = 1 - (ae^{-e^{c(t-k)}})$$

where a is the maximum extent of decay, k is the time required to reach the inflection point or maximum decay rate, and c is a fitting parameter associated with the decay rate (Section S9). The model was fit to the data using least squares regression in GraphPad Prism 8 software (La Jolla, CA) which calculated an asymmetrical 95% confidence interval for each parameter. A

two-tailed T-test was used to assess differences in the maximum extent of decay (*a*) between treatment groups. Slope regression analysis was used to determine whether trends deviate from 0. All statistical analyses were performed using GraphPad Prism.

Supplementary Results

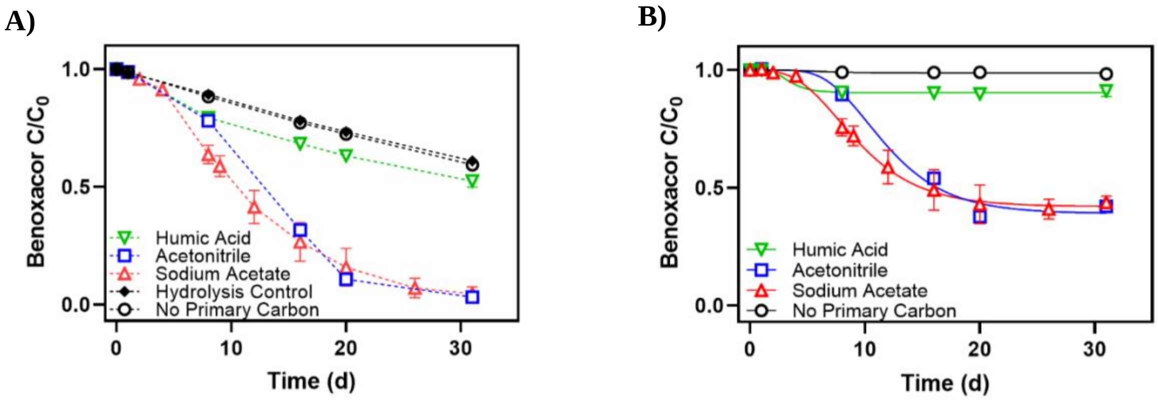

Figure S8: Normalized concentration (C/C_0) of benoxacor over time in aerobic microcosms inoculated with river sediment. All systems except for the hydrolysis control (no sediment inoculum) and the no primary carbon system (included sediment inoculum) contained a primary carbon source. A) Experimental decay curves include contributions from both biotic (microbial biotransformation) and abiotic (hydrolysis) transformation pathways. Data are depicted with a dashed connecting line. B) Normalized concentrations over time for which the contributions to benoxacor transformation from hydrolysis have been removed to illustrate the influence of microbial processes on benoxacor transformation. Solid lines represent decay curves fit with a modified, three-parameter version of the Gompertz microbial growth model (Sections S8-S9) to account for growth lag and tailing. Error bars representing one standard deviation (n=3) are present for all conditions but may be obscured by the data points.

Section S9: Gompertz Model

Table S6: Benoxacor C/C_0 in Co-metabolic systems (biotransformation and hydrolysis)

Time (d)	Aqueous			Acetonitrile			Sodium Acetate			Humic Acid		
0	1.000	1.000	1.000	1.000	1.000	1.000	1.000	1.000	1.000	1.000	1.000	1.000
1	0.988	0.990	0.985	0.988	0.988	0.988	0.989	0.985	0.986	0.985	0.985	0.986
2							0.948	0.962	0.965			
4							0.902	0.916	0.920			
8	0.882	0.884	0.884	0.780	0.793	0.768	0.593	0.651	0.667	0.783	0.795	0.802
9							0.537	0.606	0.619			
12							0.333	0.454	0.455			
16	0.768	0.771	0.776	0.316	0.318	0.317	0.171	0.312	0.314	0.674	0.680	0.695
20	0.725	0.715	0.733	0.106	0.111	0.102	0.066	0.204	0.204	0.620	0.629	0.646
26							0.024	0.100	0.086			
31	0.588	0.589	0.603	0.031	0.033	0.029	0.021	0.078	0.037	0.497	0.527	0.548

Table S7: Benoxacor Hydrolysis Control C/C_0 (no sediment inoculum)

Time (d)	Aqueous			Acetonitrile			Sodium Acetate			Humic Acid		
0	1.000	1.000	1.000	1.000	1.000	1.000	1.000	1.000	1.000	1.000	1.000	1.000
1	0.984	0.987	0.985	0.984	0.983	0.984	0.985	0.983	0.982	0.985	0.984	0.988
2							0.975	0.969	0.968			
4							0.941	0.936	0.938			
8	0.887	0.899	0.892	0.893	0.881	0.881	0.879	0.881	0.883	0.888	0.881	0.895
9							0.865	0.867	0.869			
12							0.828	0.825	0.826			
16	0.770	0.790	0.787	0.782	0.775	0.775	0.780	0.775	0.771	0.781	0.775	0.786
20	0.720	0.742	0.739	0.734	0.728	0.727	0.731	0.727	0.726	0.733	0.727	0.738
26							0.662	0.659	0.661			
31	0.596	0.620	0.615	0.610	0.611	0.610	0.598	0.610	0.615	0.613	0.610	0.615

Table S8: Benoxacor Biotransformation C/C_0 in Co-Metabolic Systems

Time (d)	Aqueous			Acetonitrile			Sodium Acetate			Humic Acid		
0	1	1	1	1	1	1	1	1	1	1	1	1
1	1.004	1.003	0.999	1.004	1.005	1.004	1.004	1.003	1.003	1	1.001	0.998
2							0.974	0.993	0.998			
4							0.961	0.98	0.982			
8	0.995	0.984	0.993	0.888	0.912	0.887	0.714	0.77	0.784	0.895	0.914	0.907
9							0.672	0.739	0.75			
12							0.505	0.629	0.629			
16	0.998	0.981	0.989	0.534	0.543	0.542	0.391	0.537	0.543	0.893	0.905	0.909
20	1.005	0.973	0.994	0.373	0.383	0.375	0.335	0.477	0.478	0.887	0.902	0.908
26							0.362	0.441	0.425			
31	0.993	0.969	0.988	0.421	0.422	0.419	0.423	0.468	0.422	0.884	0.917	0.932

To compare biotransformation rates for benoxacor in each carbon system, the data were first adjusted to remove the contributions of hydrolysis:

$$(C/C_0)_{\text{Biotransformation}} = 1 + [(C/C_0)_{\text{Experimental}} - (C/C_0)_{\text{Hydrolysis Control}}] \qquad \textbf{S9}$$

The resulting data shape produced a typical microbial degradation curve, with an initially short lag period (1-3 d) and subsequent period of exponential decay (4-10 d) that was followed by a decline in decay rate (11-20 d) and then a stationary phase (20-31 d). A modified three-parameter Gompertz model was fit to the data to determine the time to reach the maximum transformation rate; the maximum kinetic rate of transformation was determined from the model slope at the inflection point.

Based on the Gompertz equation parameters (**Table S9**), the estimated time to 50% decay can be calculated by the following equation:

$$k + \frac{\ln\left(-\ln\left(\frac{1-y}{a}\right)\right)}{c} = x \qquad \textbf{S10}$$

Where y = 50% and k, c, and a are Gompertz parameters provided in **Table S9**. The calculated time to achieve 50% decay is also present in **Table S9**.

Table S9: Gompertz parameters for microbial biotransformation of benoxacor in the presence of various primary carbon sources

Gompertz parameter	Aqueous	Acetonitrile	Sodium Acetate	Humic Acid
Total biological decay (%), *a*	1.2 (± 1.6)	60.97 (± 2.5)	58.0 (± 4.5)	9.6 (± 1.3)
Time to inflection point (d), *k*	5.2 (± 2.4)	10.2 (± 0.3)	7.7 (± 0.4)	3.0 (± 2.2)
Calculated time to 50% decay (d)	N/A[a]	16.4	14.9	N/A[a]

[a]NA = does not achieve 50% decay over the course of the 31-day experiment.

Table S10: P-values for two-tailed T-test of Gompertz parameter a, total biological decay, in microcosms containing various primary carbon sources.

**Treatment 1**	**Treatment 2**	**p-value**	**Significant?**
Aqueous	Acetonitrile	<0.0001	Yes
Aqueous	Sodium Acetate	<0.0001	Yes
Aqueous	Humic Acid	0.0023	Yes
Acetonitrile	Sodium Acetate	0.3761	No
Acetonitrile	Humic Acid	<0.0001	Yes
Sodium Acetate	Humic Acid	<0.0001	Yes

Table S11: log K_{ow} values for select chloroacetamide herbicides

Compound	log k_{ow} parent[5,6]	log k_{ow} ESA metabolite[7]	log k_{ow} OA metabolite[†]
Acetochlor	3.03	1.82	2.81
Alachlor	3.52	1.82	2.89
Metolachlor	3.13	1.69	2.51
Propachlor	2.18	0.87	1.61

[†]Estimated by ChemDraw software

The hydrolysis rate constant in the abiotic controls (0.016 d^{-1} at pH 7.4) was within 20% of the rate constant we previously reported for benoxacor hydrolysis under neutral (0.013 d^{-1} at pH 7.0) conditions; differences in rate constant are likely due to pH differences. The half-life of benoxacor was 41 days in hydrolysis controls; considering only biotransformation (i.e., no contributions from hydrolysis) in experimental systems, the estimated time to achieve 50% transformation was 16.4 days in systems containing acetonitrile as the carbon source and 14.9 days in systems containing sodium acetate as the carbon source (Equation S9, Table S9). Thus, if a labile carbon source is available to facilitate co-metabolic microbial biotransformation in surface water, groundwater, or sediment systems, the microbial biotransformation of benoxacor is likely of greater importance to its long-term fate than hydrolytic processes.

Table S12: Formulas, molecular weights, and retention times for major and minor peaks present in benoxacor biotransformation samples measured on Orbitrap LC-MS. Peaks were designated as "major" or "minor" based on Log_2 fold change, molecular weight, and peak shape/symmetry.

RT (min)	Formula	Molecular Weight	Major or Minor peak	Log_2 fold change	Proposed Structure
3.9	**C16 H20 N2 O5 S**	**352.108**	**Major (Benox-352)**	**9.3**	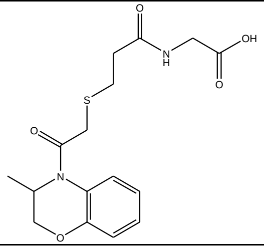
8.1	**C15 H20 N2 O3 S**	**308.119**	**Major**	**11.5**	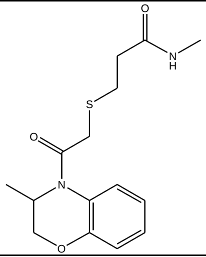

9.0	**C11 H13 N O2**	**191.095**	**Major**	**8.3**	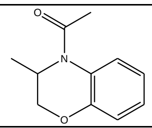
9.8	C8 H7 N O	133.053	Minor	7.84	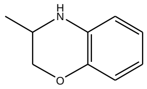
	C6 H7 N O2	107.037	Minor	5.85	
	**C9 H11 N O**	**149.084**	**Major (hydrolysis product)**	**10.56**	
	C6 H6 N O	108.045	Minor	7.32	
	C9 H10 N O	148.07636	Minor	8.78	
	C9 H9 N O	147.06842	Minor	4.38	
10.7	**C11 H12 Cl N O2**	**225.0555**	**Major (Monochloro-benoxacor)**	**7.6**	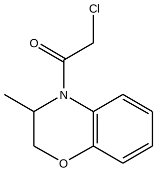
	C10 H11 N O	161.08383	Minor	5.88	
	C11 H11 N O2	189.07881	Minor	9.54	
	C2 H5 Cl N2 O2 S	155.97653	Minor	5.62	
10.9	**C12 H15 N O2 S**	**237.0822**	**Major**	**10.0**	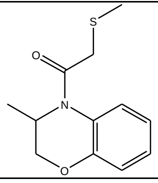
	C9 H11 N O	149.08397	Minor	9.25	
	C10 H11 N O	161.08386	Minor	6.52	
12.9	C12 H13 N O2 S	235.0665	Minor	8.5	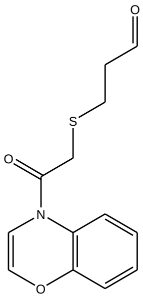
	C8 H7 N O	133.0528	Minor	4.2	
	C10 H11 N O	161.084	Minor	8.6	
	**C13 H13 N O3 S**	**263.0614**	**Major (Benox-263)**	**7.1**	
	C10 H11 N O S	193.0561	Minor	5.5	
13.5	**C11 H11 N O2 S**	**221.0509**	**Major**	**6.5**	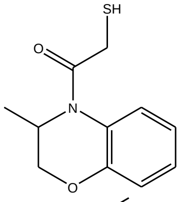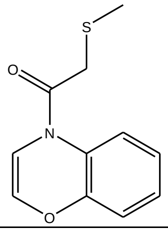
	**C11 H13 N O2 S**	**223.0665**	**Major**	**6.1**	
	C11 H9 N O2	187.06314	Minor	5.81	
	C10 H11 N O	161.08388	Minor	5.15	

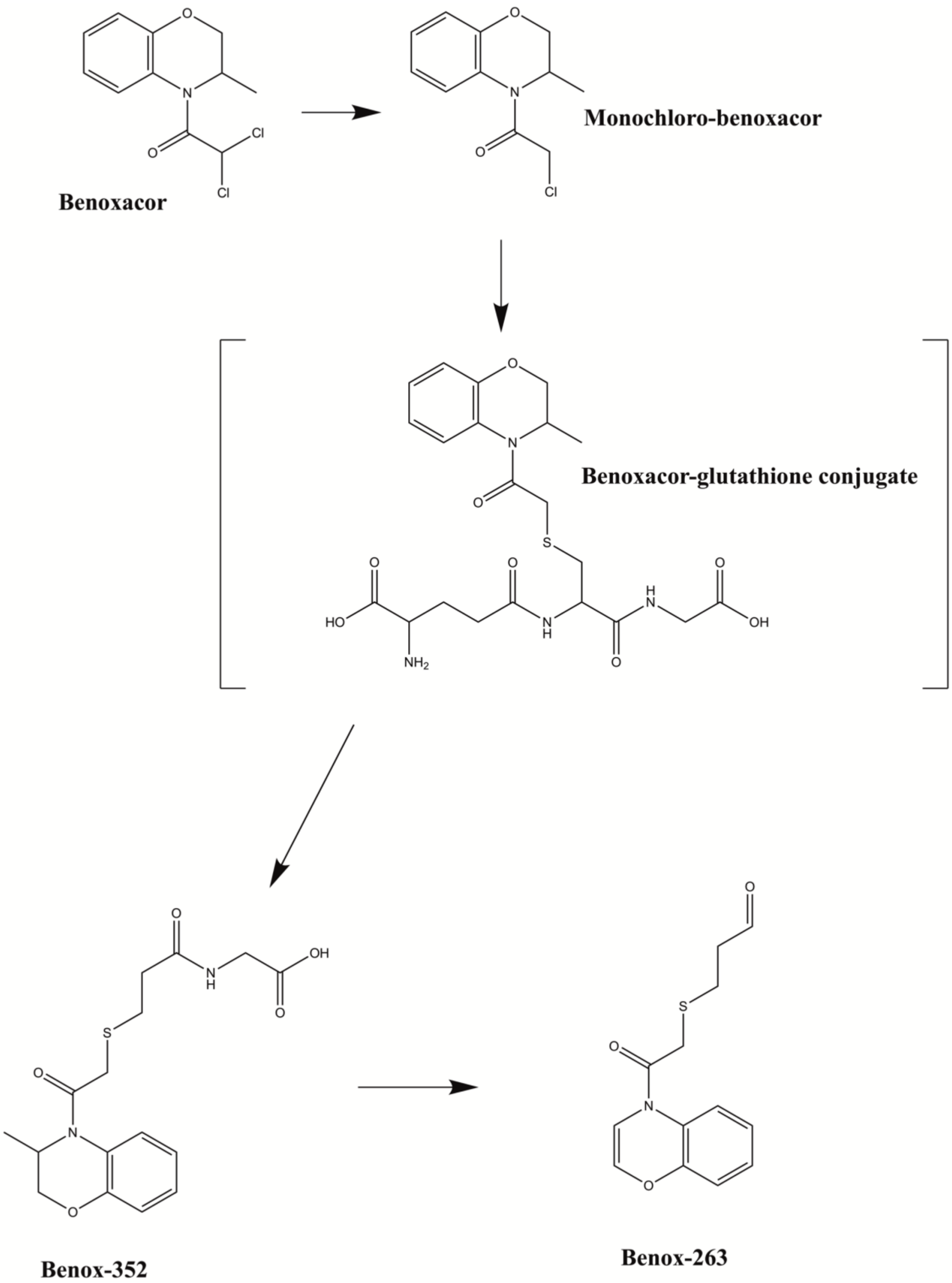

Scheme S1: Proposed glutathione conjugation microbial biotransformation pathway between glutathione and the dichloroacetamide safener benoxacor to form sulfonated metabolites. The compound in brackets [ ] is an inferred intermediate to the observed compounds.

Biotransformation Products of Benoxacor

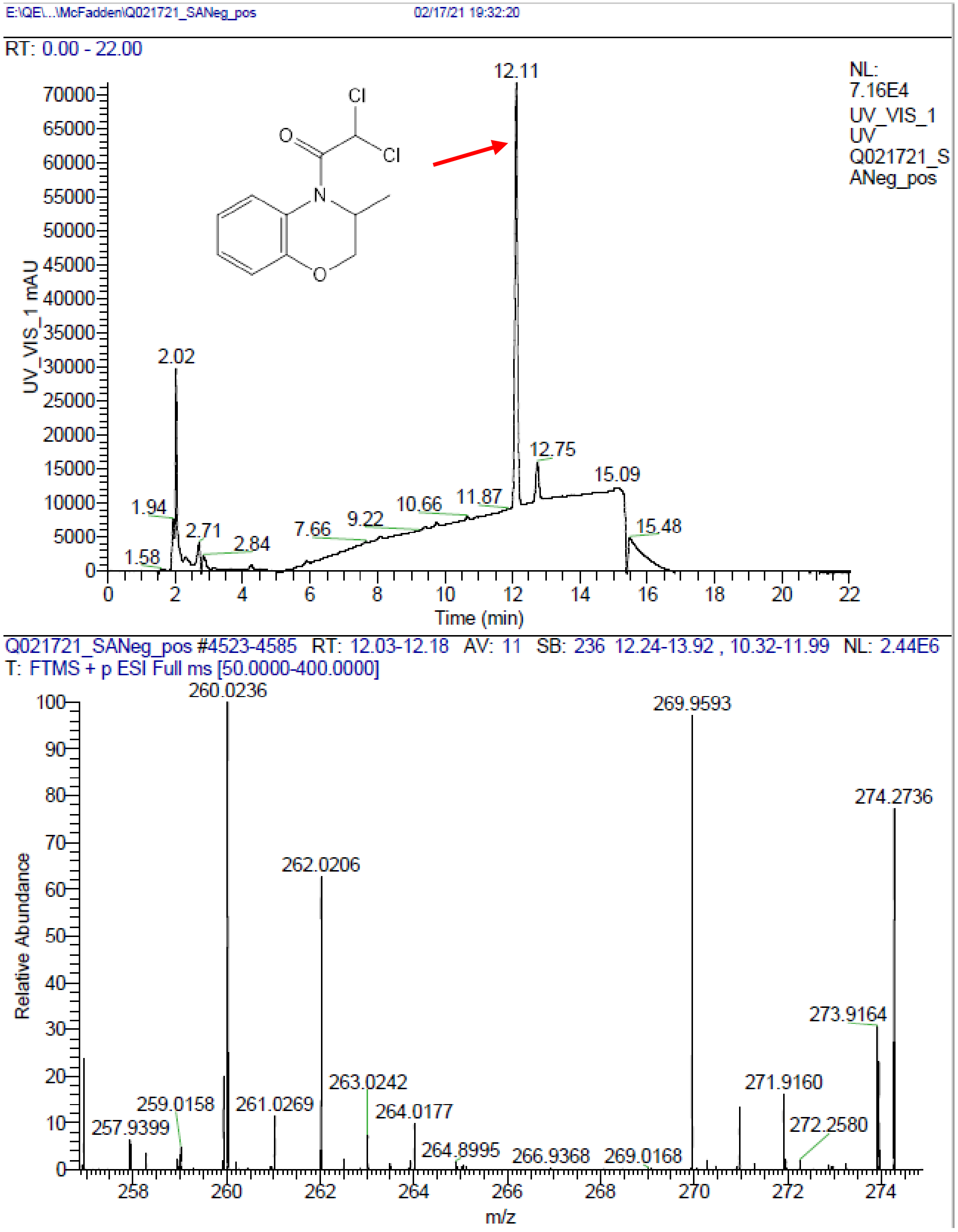

Figure S9: Orbitrap LC Chromatogram (UV 220 nm) and HRMS spectrum of the parent compound, benoxacor.

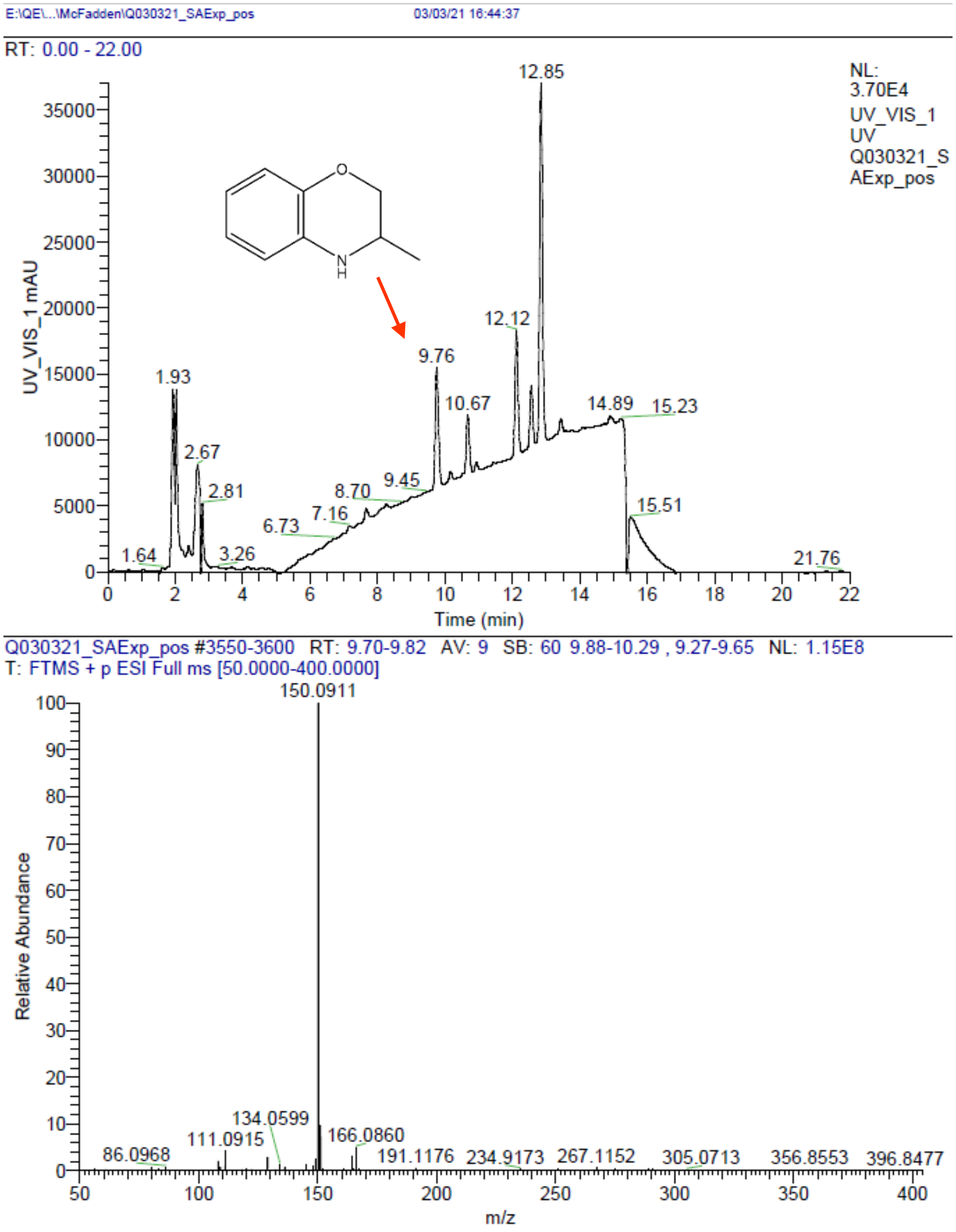

Figure S10: LC Chromatogram and HRMS spectrum of the benoxacor experimental system with sodium acetate after 24 days, showing the benoxacor hydrolysis product previously identified as 3-methyl-3,4-dihydro-2H-1,4-benzoxazine.

A)

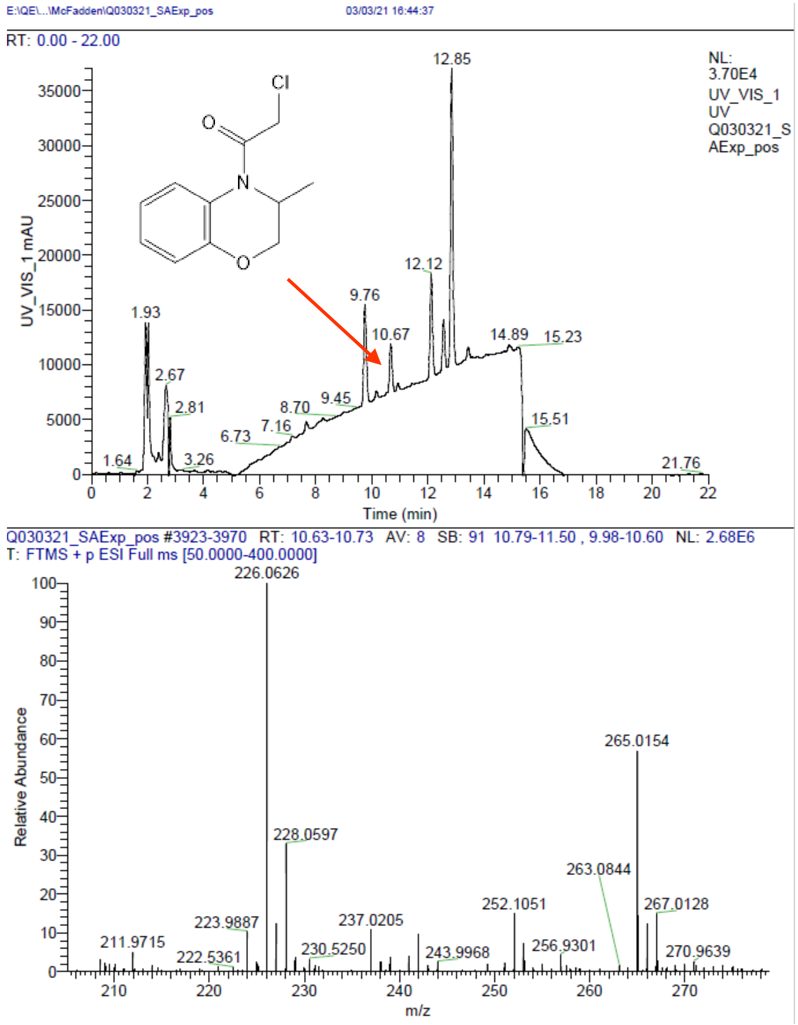

B)

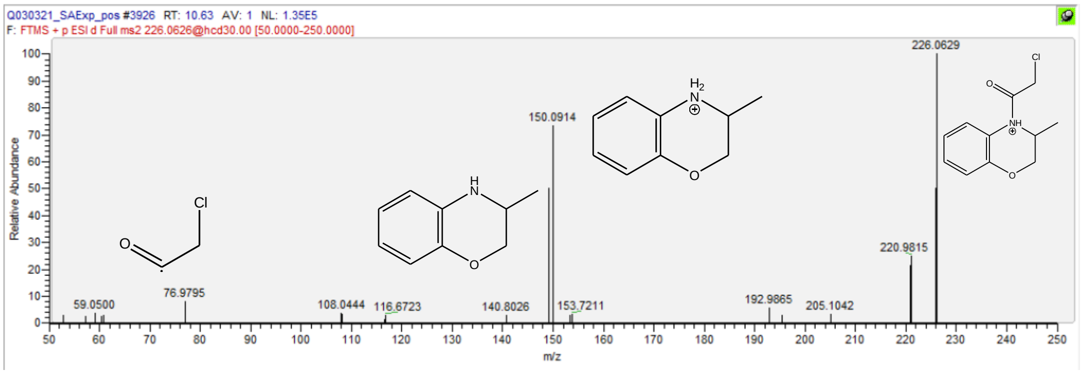

Figure S11: A) LC Chromatogram and HRMS spectrum of the benoxacor experimental system with sodium acetate after 24 days, showing the monochloro-benoxacor product. Monochloro-benoxacor is observed at RT 10.7 min, and the HRMS spectrum shows a chlorine isotope signature for a product with $[H+M]^+$ 226 that includes one chlorine atom. B) LC-MS/MS fragmentation spectra of the monochloro-benoxacor product peak at retention time (RT) 10.63 minutes with proposed fragment structures.

A)

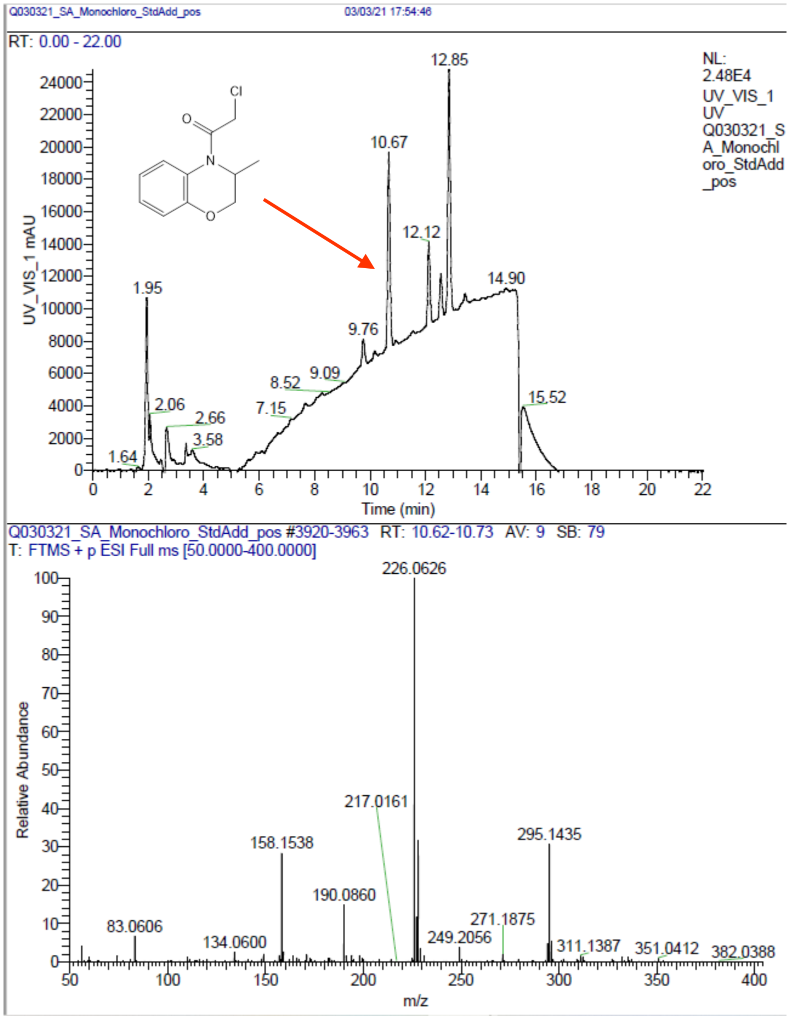

B)

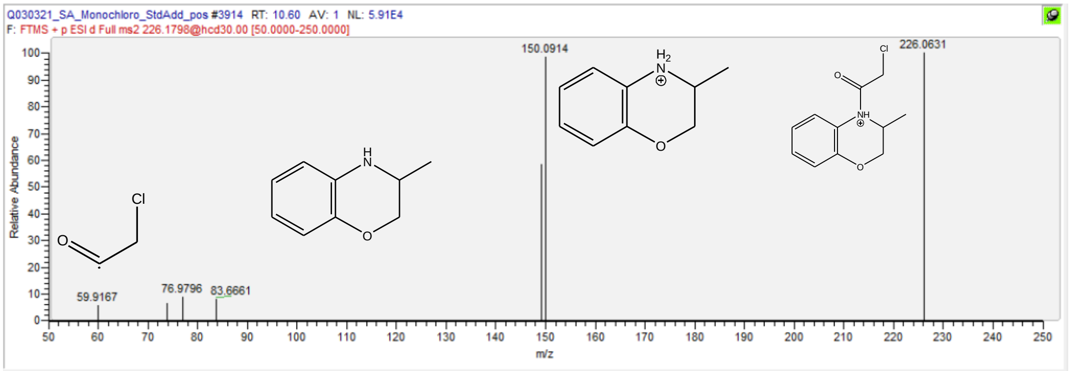

Figure S12: A) LC Chromatogram and HRMS spectrum of the benoxacor experimental system with sodium acetate after 24 days, spiked with monochloro-benoxacor. Compared to Figure S11, this confirms the structure of the product at RT 10.67 as monochloro-benoxacor with Level 1 confidence, due to matches in retention time, MS, and MS/MS. Y-axis values differ from Fig. S11 because of sample dilution with a larger volume of a low-concentration monochloro-benoxacor stock. B) LC-MS/MS fragmentation spectra of the monochloro-benoxacor product peak at retention time (RT) 10.63 minutes with proposed fragment structures.

A)

B)

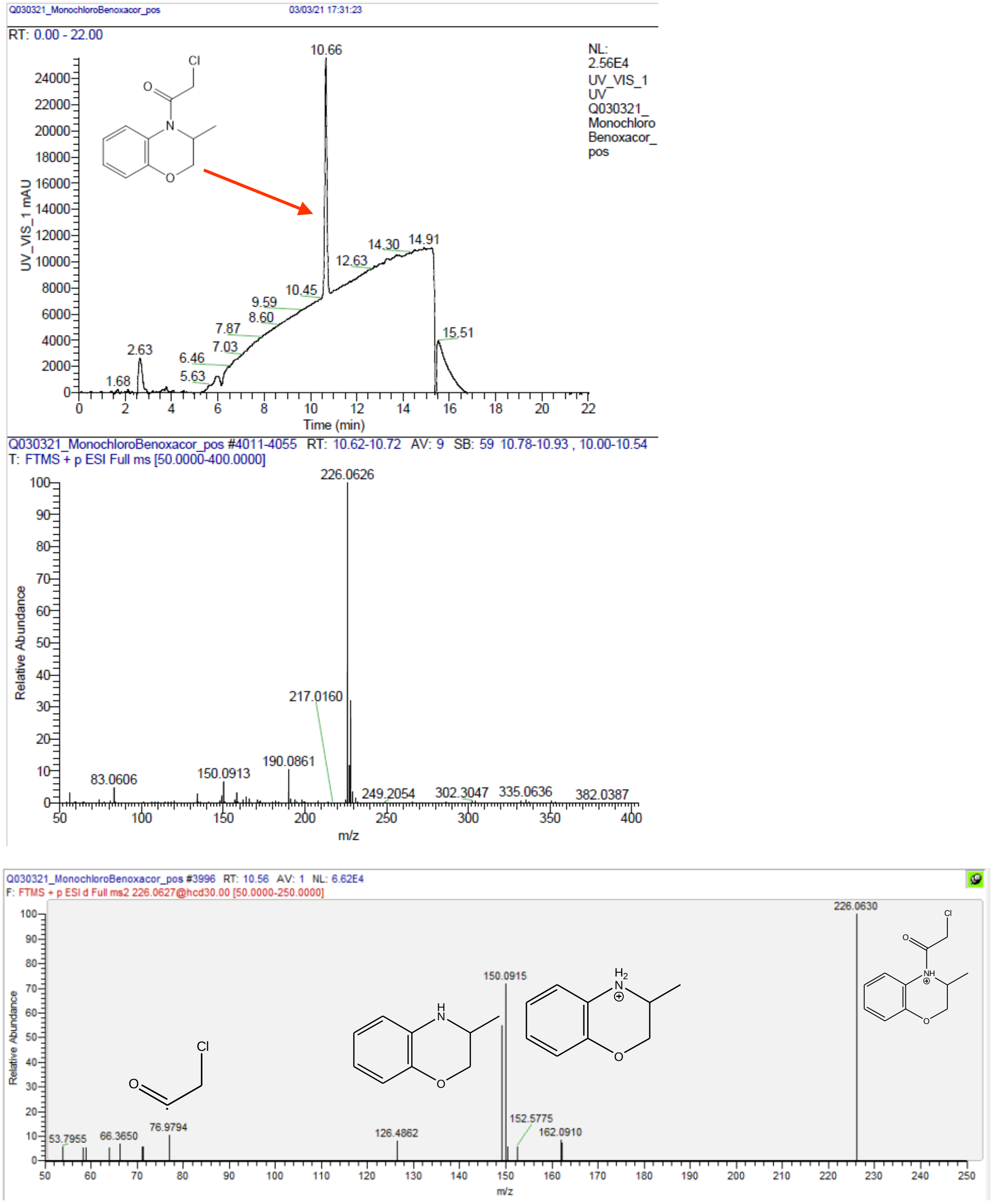

Figure S13: A) LC Chromatogram and HRMS spectrum of the monochloro-benoxacor stock. B) LC-MS/MS fragmentation spectra of the monochloro-benoxacor product peak at retention time (RT) 10.63 minutes with proposed fragment structures.

A)

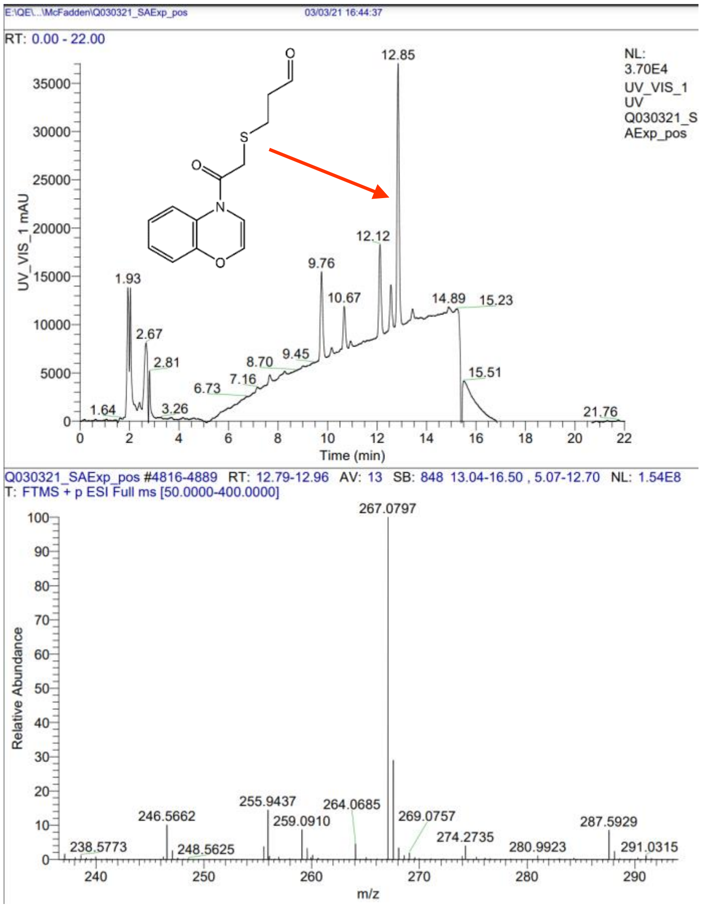

B)

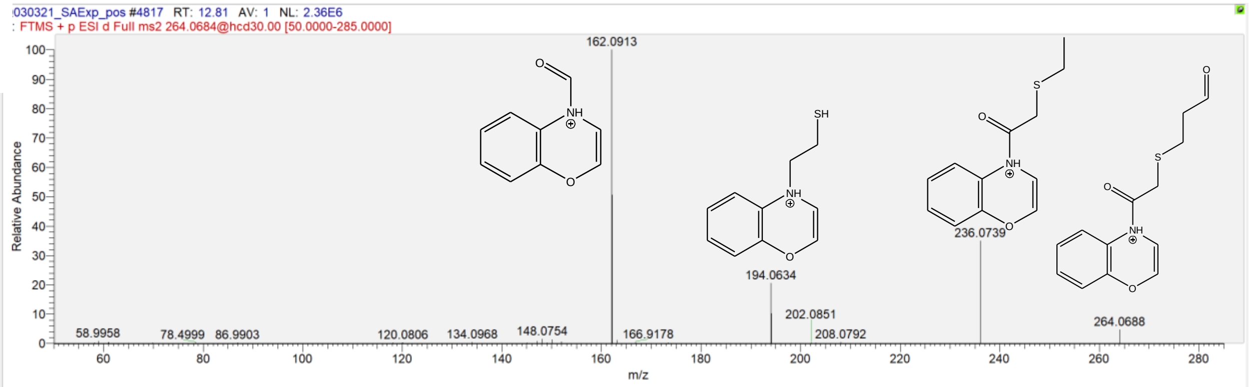

Figure S14: A) LC Chromatogram and HRMS spectrum of the benoxacor experimental system with sodium acetate after 24 days, showing the cysteine conjugate, Benox-263. The LC chromatogram is filtered by mass, showing that the mass of interest is entirely within the peak at 12.85 minutes. While the HRMS spectrum shows only a small peak for the mass of interest (264.0685), the peak at 267 is related to Benox-263 via MS/MS and is likely a fragment adduct. MS/MS analysis and masses of minor products which suggest close structural relationships (**Table S12**) lend confidence to this product structure. B) LC-MS/MS fragmentation spectra of Benox-263 at retention time (RT) 12.81 minutes with proposed fragment structures.

A)

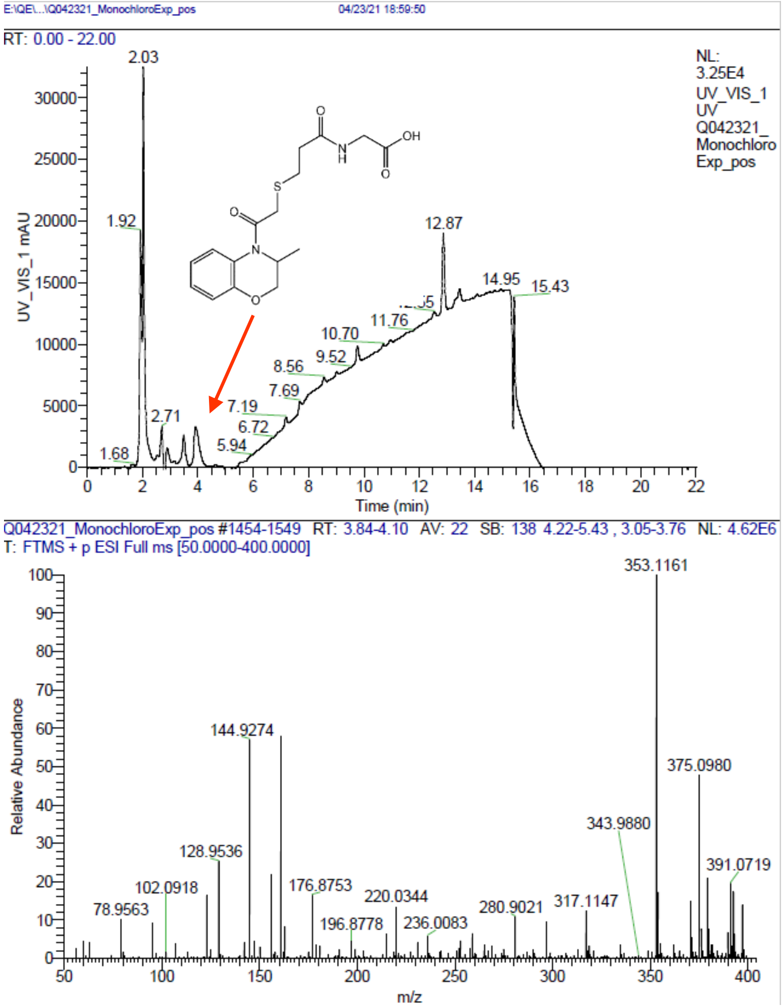

B)

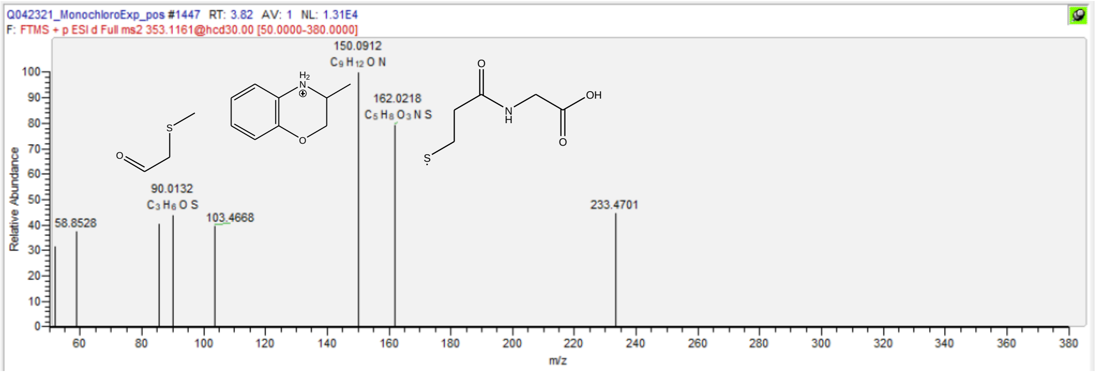

Figure S15: A) LC Chromatogram and HRMS spectrum of the monochloro-benoxacor experimental system, showing Benox-352. B) LC-MS/MS fragmentation spectra of the Benox-352 product peak at RT 3.82 minutes with proposed fragment structures. B) LC-MS/MS fragmentation spectra of Benox-352 at retention time (RT) 3.82 minutes with proposed fragment structures.

A)

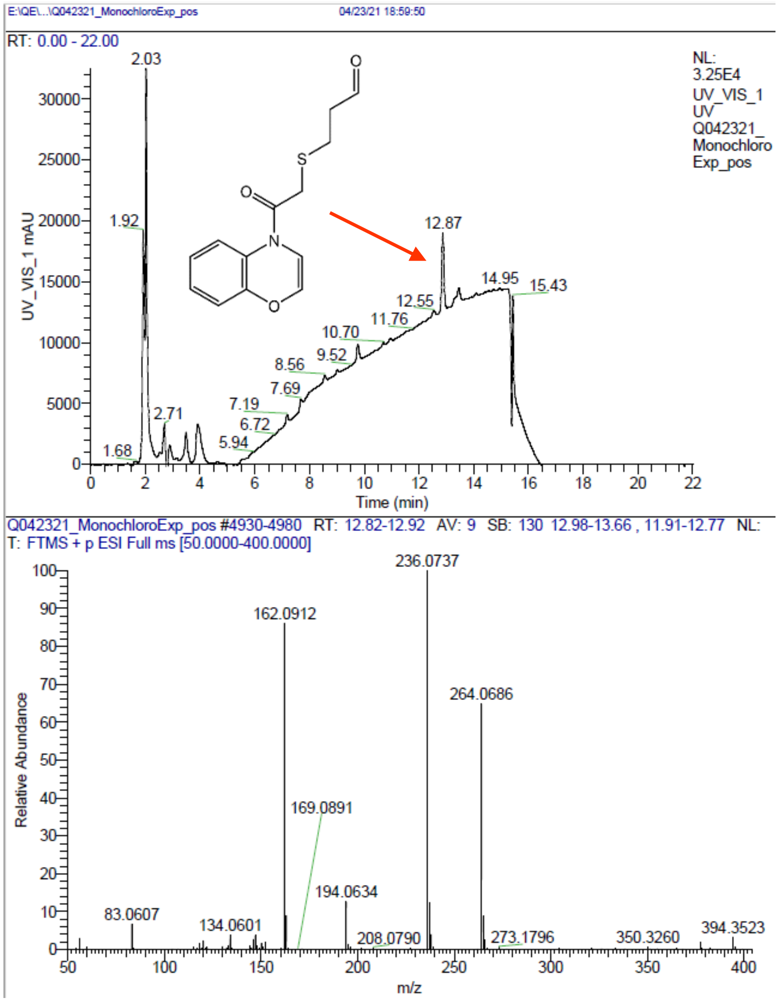

B)

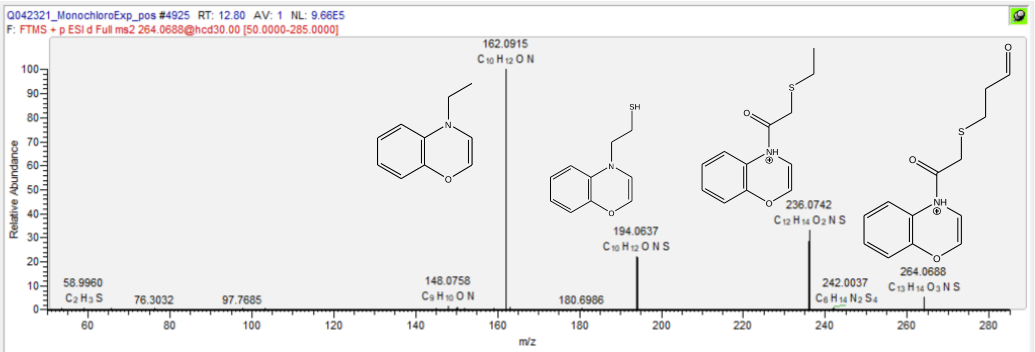

Figure S16: A) LC Chromatogram and HRMS spectrum of the monochloro-benoxacor experimental system, showing Benox-263. B) LC-MS/MS fragmentation spectra of the Benox-263 product peak at RT 12.80 minutes with proposed fragment structures.

Biotransformation Products of Dichlormid

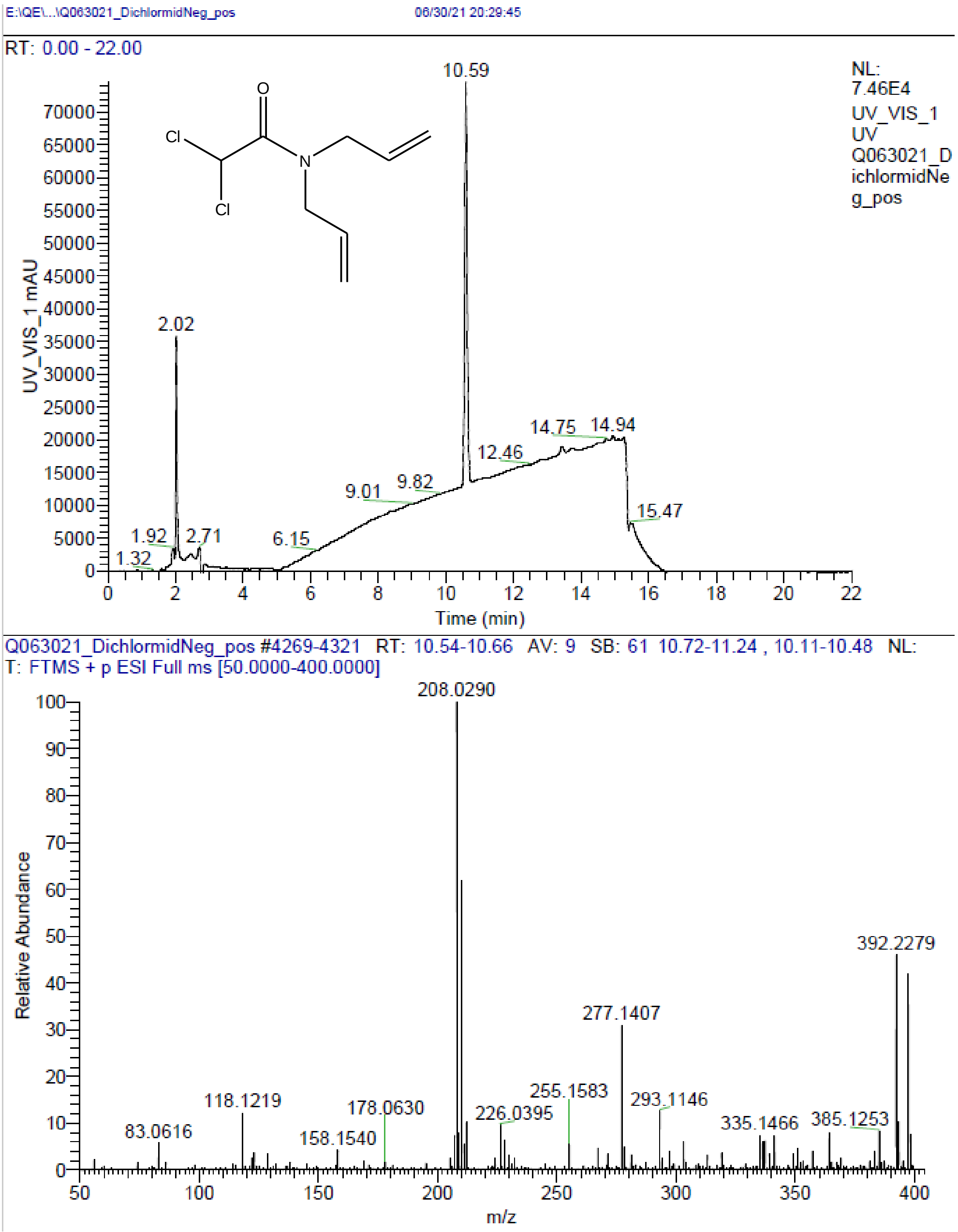

Figure S17: LC Chromatogram and HRMS spectrum of the dichlormid starting material in ESI-positive mode.

A)

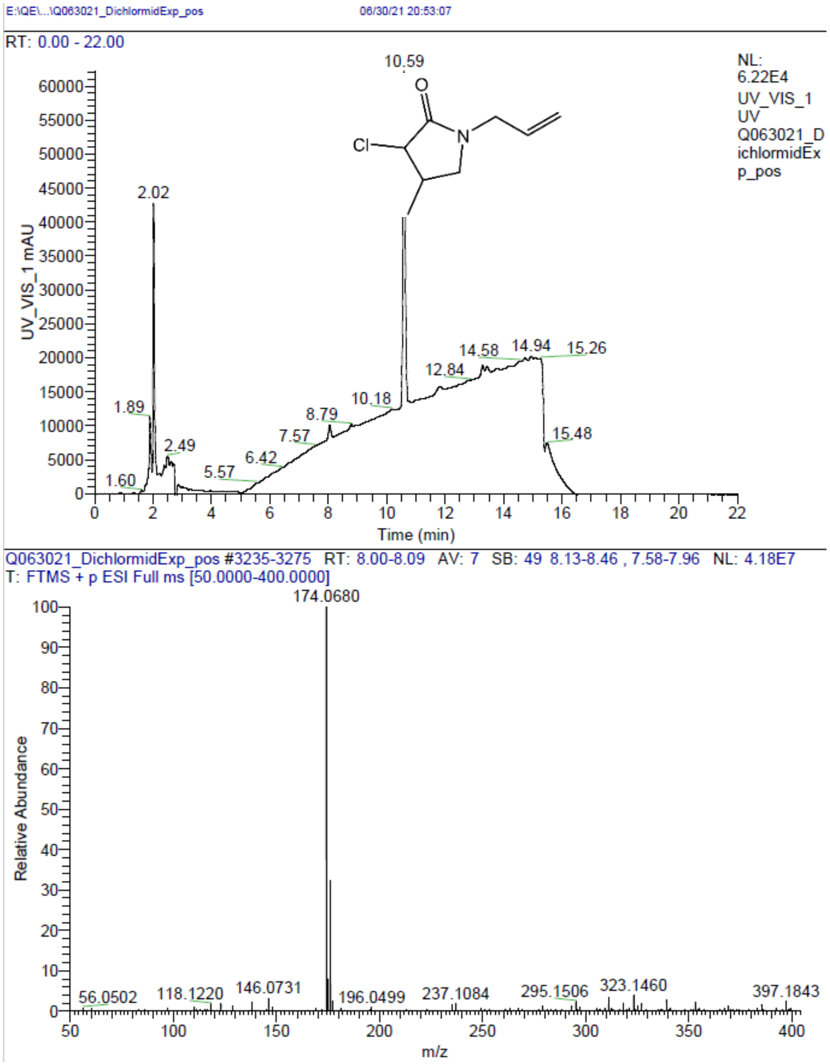

B)

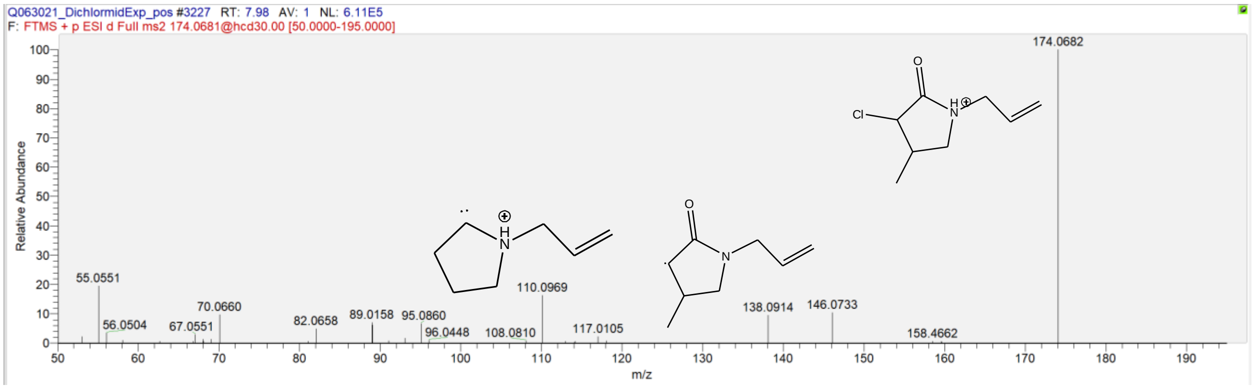

Figure S18: LC Chromatogram and HRMS spectrum of the dichlormid microbial biotransformation product Dich-173 in ESI-positive mode. B) LC-MS/MS fragmentation spectra of the Dich-173 product peak at RT 8.0 minutes with proposed fragment structures.

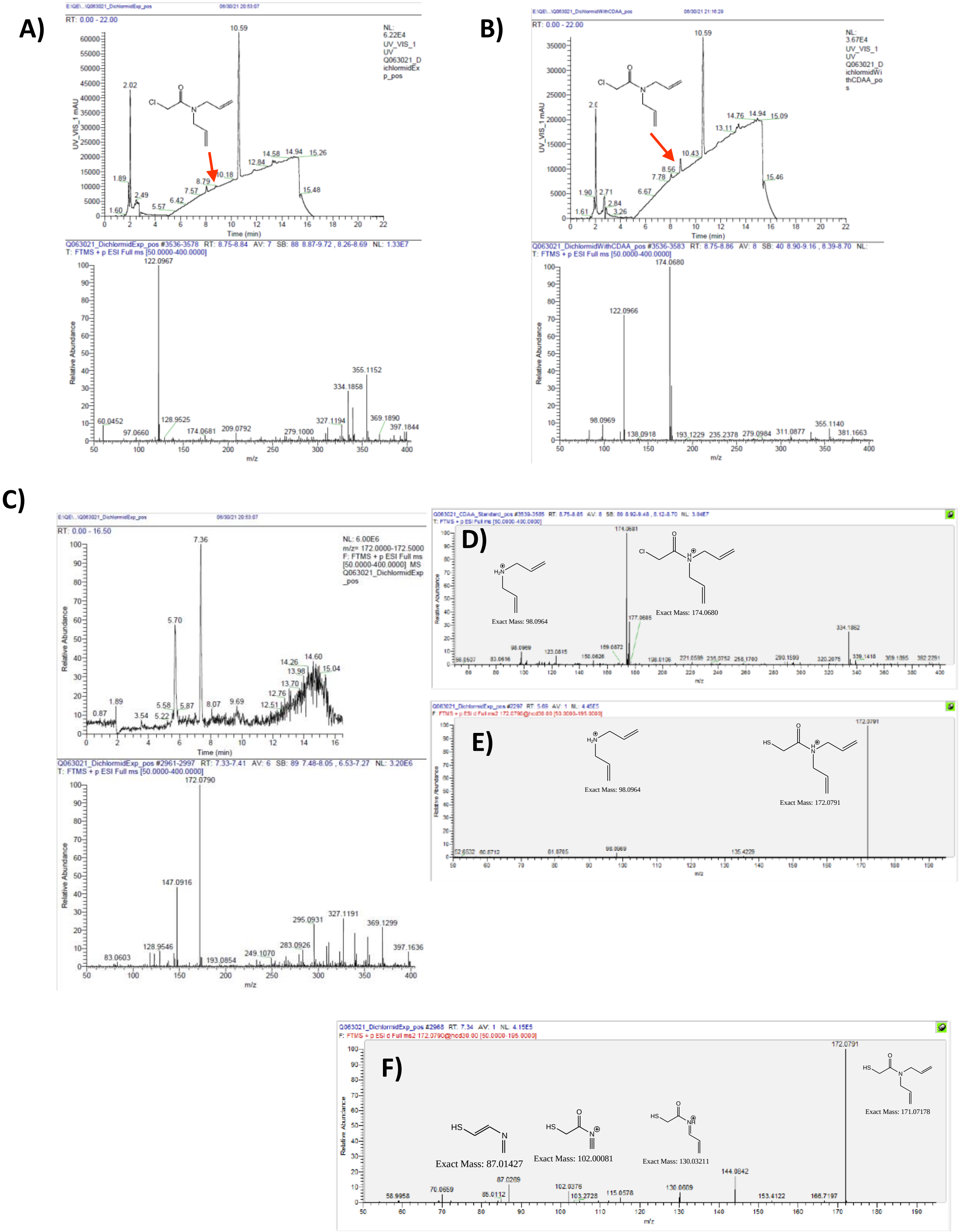

A)
RT: 0.00 - 22.00
UV_VIS_1 mAU
Time (min)
Q063021_DichlormidExp_pos
T: FTMS + p ESI Full ms [50.0000-400.0000]
Relative Abundance
m/z
122.0967
355.1152
334.1858
B)
RT: 0.00 - 22.00
UV_VIS_1 mAU
Time (min)
Q063021_DichlormidWithCDAA_pos
T: FTMS + p ESI Full ms [50.0000-400.0000]
Relative Abundance
m/z
174.0680
122.0966
C)
RT: 0.00 - 16.50
Relative Abundance
Time (min)
Q063021_DichlormidExp_pos
T: FTMS + p ESI Full ms [50.0000-400.0000]
172.0790
147.0916
m/z
D)
Exact Mass: 98.0964
Exact Mass: 174.0680
E)
Exact Mass: 98.0964
Exact Mass: 172.0791
F)
Exact Mass: 87.01427
Exact Mass: 102.00081
Exact Mass: 130.03211
Exact Mass: 171.07178
172.0791
144.0842
m/z

Figure S19: (A) LC Chromatogram and HRMS spectrum of the dichlormid microbial biotransformation product CDAA in ESI-positive mode at 8.8 minutes. This product had a small peak area that did not ionize well; as such, MS2 data is not available. For that reason, other MS evidence is presented to support identification of CDAA. (B) LC Chromatogram and HRMS spectrum of the dichlormid microbial biotransformation product CDAA in ESI-positive mode at 8.8 minutes with addition of a purchased CDAA standard. (C) LC Chromatogram of the dichlormid microbial biotransformation system, filtered by mass (m/z = 172-172.5), indicating the presence of products with RT = 5.7 and 7.4. The HRMS spectrum of the product with RT 7.4 minutes is provided. Based on data presented in Figure S23, these products result from biotransformation of CDAA and could explain why the CDAA peak area was low in panel A. (D) LC-MS/MS fragmentation spectra of the experimental system spiked with a CDAA standard addition at RT 8.8 minutes with fragment structures. (E) LC-MS/MS fragmentation spectra of the CDAA product peak at RT 5.7 minutes with proposed fragment structures. (F) LC-MS/MS fragmentation spectra of the CDAA product peak at RT 7.4 minutes (identified as the product CD-171).

A)

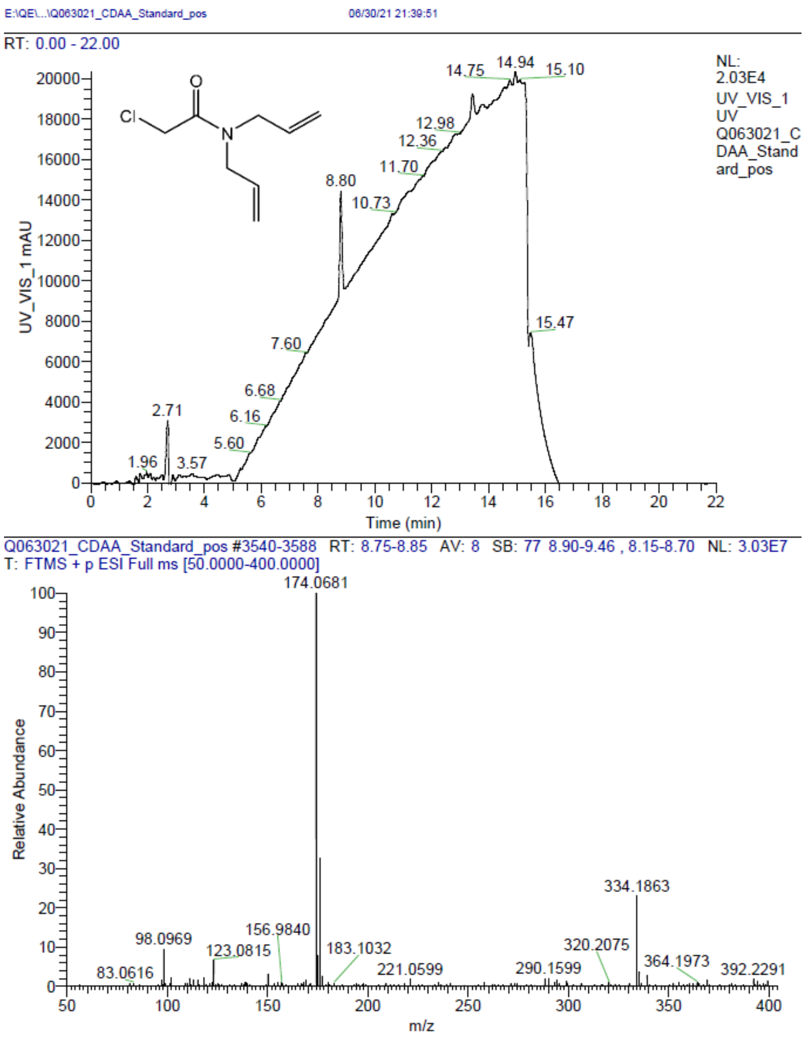

B)

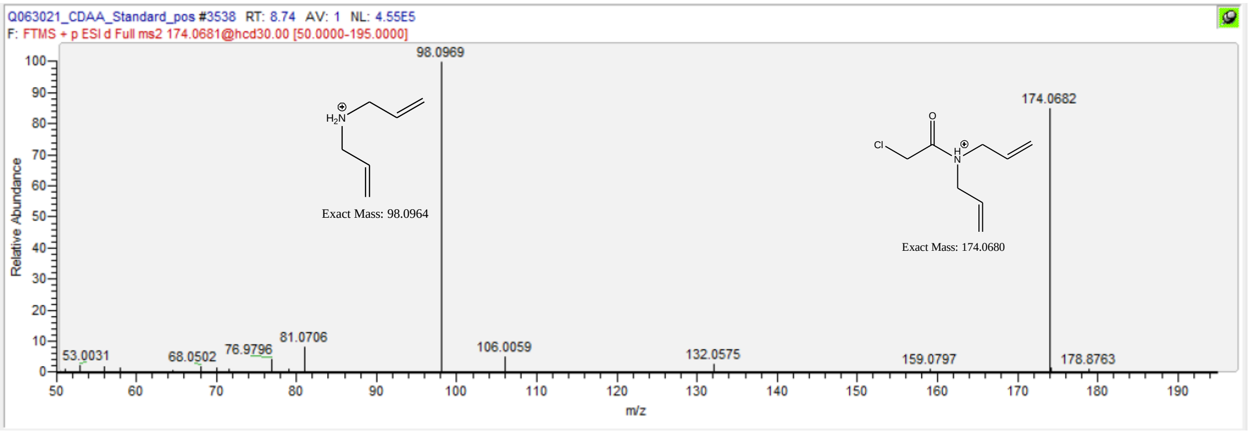

Figure S20: LC Chromatogram and HRMS spectrum of the CDAA standard in ESI-positive mode

Biotransformation Products of CDAA

A)

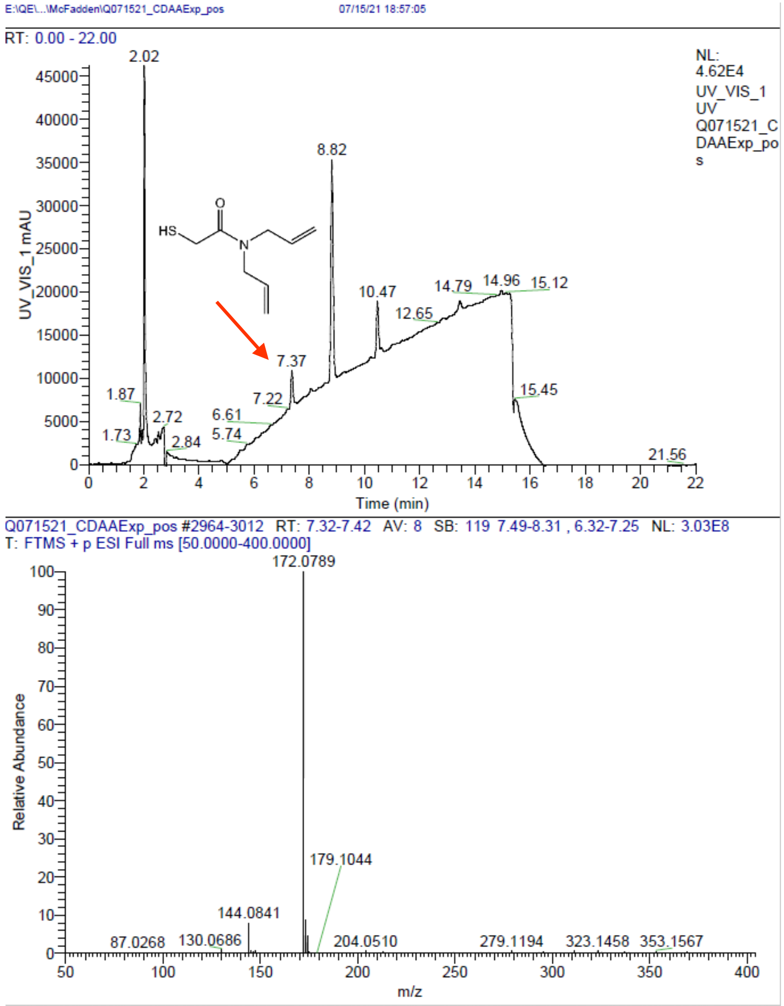

B)

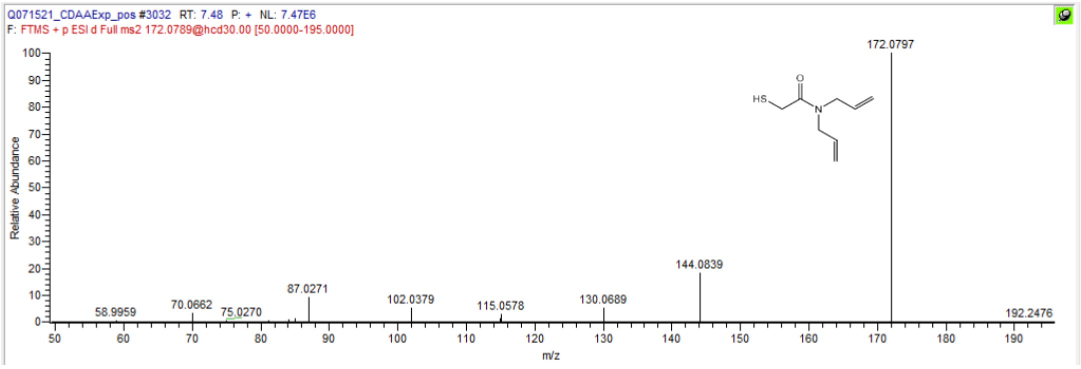

Figure S21: LC Chromatogram and HRMS spectrum of the CDAA microbial biotransformation product CD-171 in ESI-positive mode. This product has the same retention time and exact mass as the CDAA product shown in Figure S19. B) LC-MS/MS fragmentation spectra of the CD-171 product peak at RT 7.4 minutes.

A)

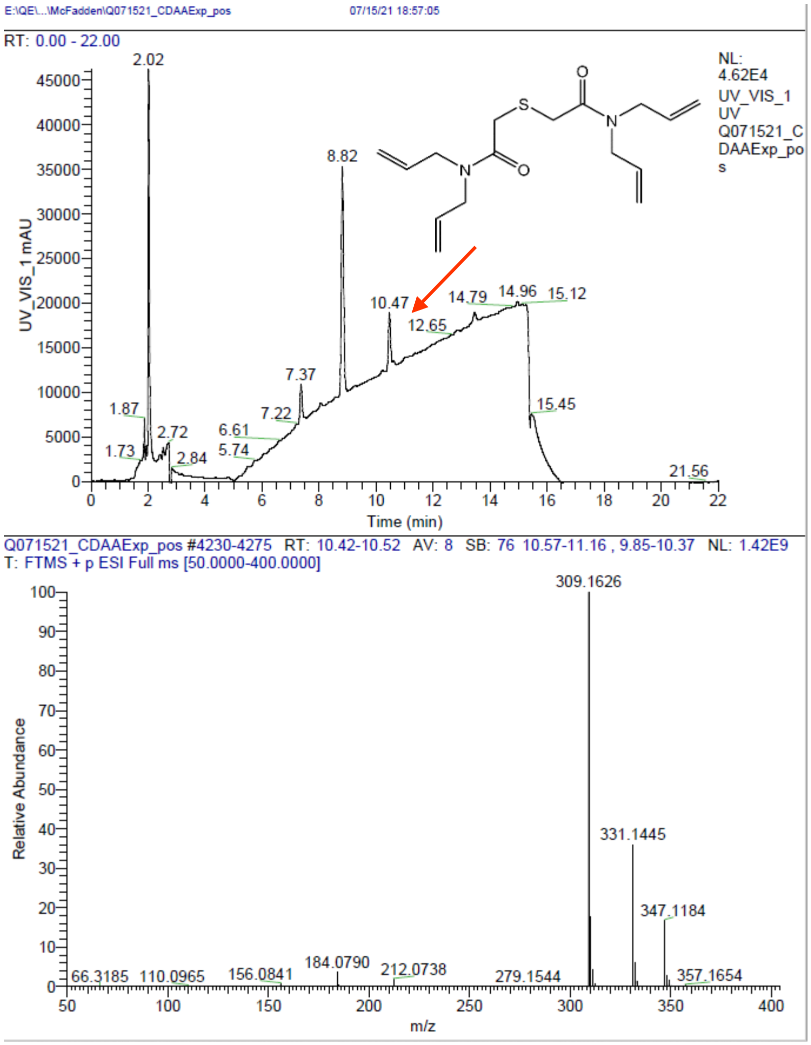

B)

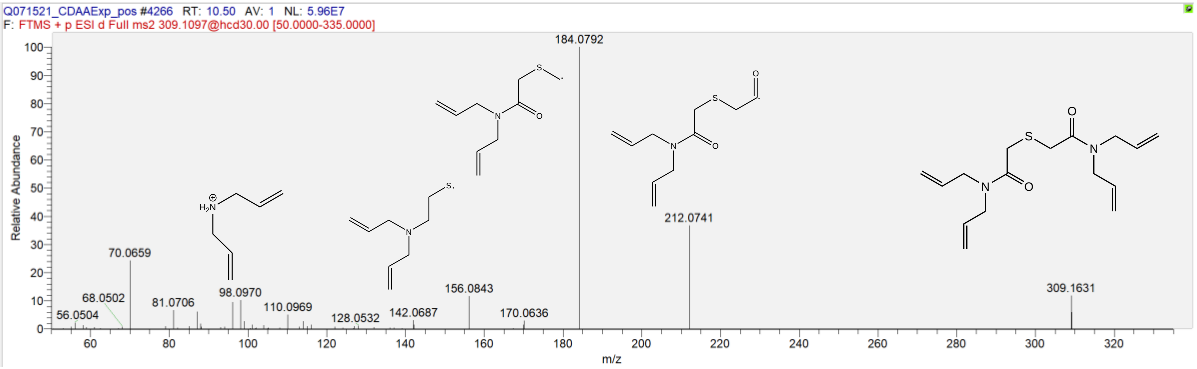

Figure S22: LC Chromatogram and HRMS spectrum of the CDAA microbial biotransformation product CD-308 in ESI-positive mode at RT 10.74 min. B) LC-MS/MS fragmentation spectra of the CD-308 product peak at RT 10.5 minutes with proposed fragment structures.

A)

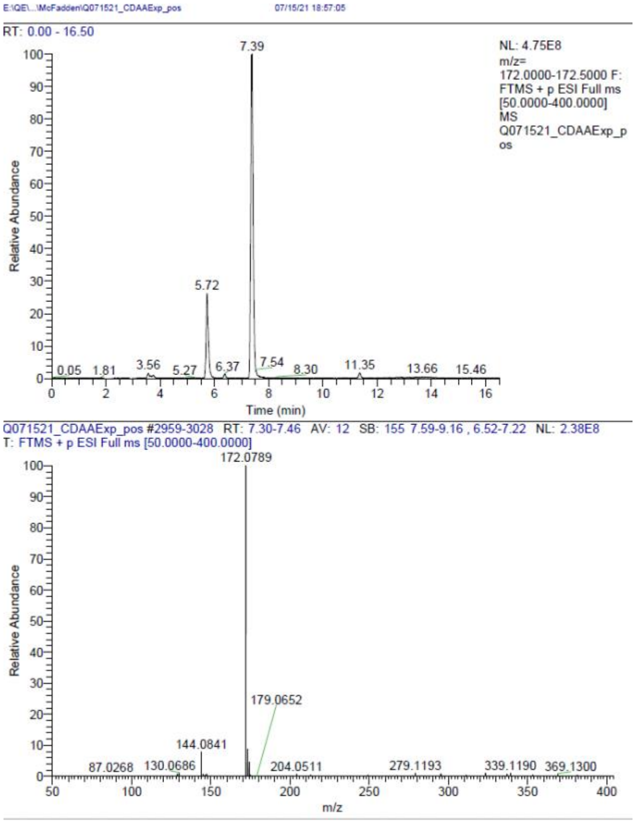

B)

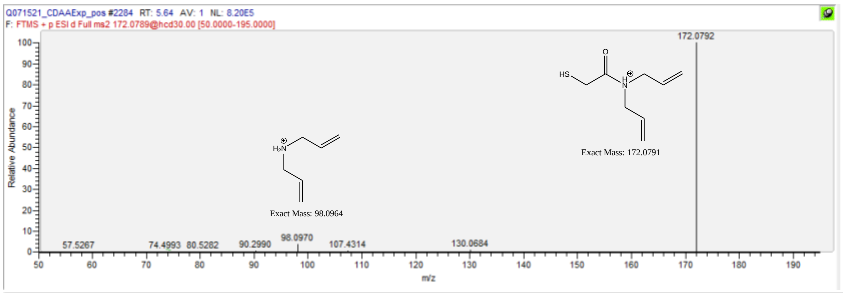

C)

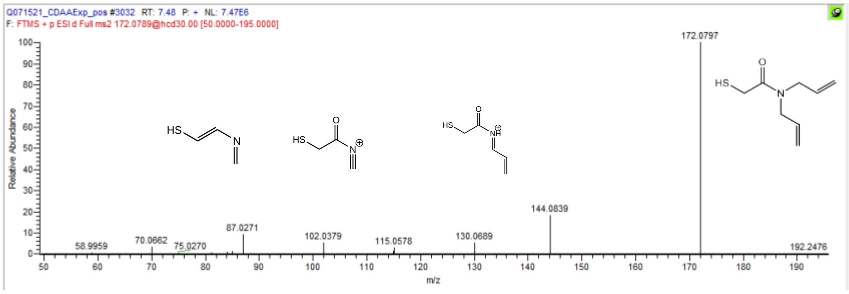

Figure S23: A) LC Chromatogram of the CDAA microbial biotransformation system, filtered by mass (m/z = 172-172.5), indicating the presence of CDAA products with RT = 5.7 and 7.4. These two peaks are consistent with those shown for the dichlormid biotransformation system in Figure S19, and the peak at 7.4 is indicative of the CD-171 product, based on the RT and m/z. (Figure S21). These results suggest that for the dichlormid biotransformation system, the CDAA peak was small because CDAA rapidly transforms into CD-171 and other products. B) LC-MS/MS fragmentation spectra of the filtered mass peak at RT 5.7 minutes with proposed

fragment structures. This spectra is very similar to that in Fig. 19E . C) LC-MS/MS fragmentation spectra of the filtered mass peak at RT 7.4 minutes. This MS2 spectra for this RT is very similar to that in Fig. S19 F.

References

(1) Ghoshal, S.; Ramaswami, A.; Luthy, R. G. Biodegradation of Naphthalene from Coal Tar and Heptamethylnonane in Mixed Batch Systems. *Environ. Sci. Technol.* **1996**, *30* (4), 1282–1291 DOI: 10.1021/es950494d.

(2) Wiesebron, L. E.; Steiner, N.; Morys, C.; Ysebaert, T.; Bouma, T. J. Sediment Bulk Density Effects on Benthic Macrofauna Burrowing and Bioturbation Behavior . *Frontiers in Marine Science* . 2021.

(3) Zhi, H.; Mianecki, A. L.; Kolpin, D. W.; Klaper, R. D.; Iwanowicz, L. R.; LeFevre, G. H. Tandem Field and Laboratory Approaches to Quantify Attenuation Mechanisms of Pharmaceutical and Pharmaceutical Transformation Products in a Wastewater Effluent-Dominated Stream. *Water Res.* **2021**, *203*, 117537 DOI: https://doi.org/10.1016/j.watres.2021.117537.

(4) Kral, A. E.; Pflug, N. C.; McFadden, M. E.; LeFevre, G. H.; Sivey, J. D.; Cwiertny, D. M. Photochemical Transformations of Dichloroacetamide Safeners. *Environ. Sci. Technol.* **2019**, *53* (12), 6738–6746 DOI: 10.1021/acs.est.9b00861.

(5) Tomlin, C.; British Crop Protection Council. *The Pesticide Manual: A World Compendium.*, Fifteenth.; Tomlin, C., Ed.; Alton : BCPC, 2009.

(6) Hansch, C.; Leo, A.; Hoekman, D. H. *Exploring QSAR: Fundamentals and Applications in Chemistry and Biology*; American Chemical Society: Washington, DC, 1995; Vol. 557.

(7) US EPA. Estimation Programs Interface Suite for Microsoft Windows, v 4.11. United States Environmental Protection Agency: Washington, DC 2014.
